# Identifying properties of pattern completion neurons in a computational model of the visual cortex

**DOI:** 10.1371/journal.pcbi.1011167

**Published:** 2023-06-06

**Authors:** Casey M. Baker, Yiyang Gong

**Affiliations:** 1 Department of Biomedical Engineering, Duke University, Durham, North Carolina, United States of America; 2 Department of Neurobiology, Duke University, Durham, North Carolina, United States of America; Chinese Academy of Sciences, CHINA

## Abstract

Neural ensembles are found throughout the brain and are believed to underlie diverse cognitive functions including memory and perception. Methods to activate ensembles precisely, reliably, and quickly are needed to further study the ensembles’ role in cognitive processes. Previous work has found that ensembles in layer 2/3 of the visual cortex (V1) exhibited pattern completion properties: ensembles containing tens of neurons were activated by stimulation of just two neurons. However, methods that identify pattern completion neurons are underdeveloped. In this study, we optimized the selection of pattern completion neurons in simulated ensembles. We developed a computational model that replicated the connectivity patterns and electrophysiological properties of layer 2/3 of mouse V1. We identified ensembles of excitatory model neurons using K-means clustering. We then stimulated pairs of neurons in identified ensembles while tracking the activity of the entire ensemble. Our analysis of ensemble activity quantified a neuron pair’s power to activate an ensemble using a novel metric called pattern completion capability (PCC) based on the mean pre-stimulation voltage across the ensemble. We found that PCC was directly correlated with multiple graph theory parameters, such as degree and closeness centrality. To improve selection of pattern completion neurons *in vivo*, we computed a novel latency metric that was correlated with PCC and could potentially be estimated from modern physiological recordings. Lastly, we found that stimulation of five neurons could reliably activate ensembles. These findings can help researchers identify pattern completion neurons to stimulate *in vivo* during behavioral studies to control ensemble activation.

## Author summary

Neural ensembles are groups of neurons that fire together in response to stimuli. Ensembles and pattern completion may play important functional roles in cognition. For example, in the mouse visual cortex, activation of an ensemble via stimulation of just two pattern completion neurons can drive visual perception. We improved the understanding of such pattern completion properties by developing a computational model of the visual cortex that recapitulated the cortex’s structural and functional properties. We then identified ensembles in our model, repeatedly stimulated different pairs of neurons, and recorded the fraction of times that this stimulation resulted in ensemble activation. We found that neurons that strongly activated ensembles could complete patterns when the average ensemble voltage was far from the threshold. We also found that graph theory parameters could reliably predict efficient pattern completion neurons. Lastly, we developed a novel latency metric that could identify pattern completion neurons *in vivo* using modern imaging techniques.

## Introduction

Neural ensembles are groups of neurons that are consistently coactive both spontaneously and in response to stimuli [1–5]. They are found in diverse regions of the brain including the motor cortex, sensory cortices, and the hippocampus [6–10]. Neuroscientists have long believed that ensembles play a functional role in cognition, and recent experimental work has found ensembles that underlie associative memories, motor behaviors, and visual perception [6–8].

Recording and manipulating ensemble activity can help elucidate ensemble formation, activation, and function. One form of ensemble activation under study is pattern-completion, where ensembles composed of tens of neurons can be activated by the stimulation of just a few neurons [8,11–13]. Understanding the function of pattern completion neurons can help elucidate how diverse stimuli are encoded in the brain and how information is efficiently transferred across brain regions [4,14,15]. For example, it is possible that the brain transmits information to different brain regions by stimulating ensembles in these regions via synaptic connections with pattern completion neurons. Additionally, pattern completion in the visual cortex may explain how organisms can recognize partially occluded objects [16,17].

Methodical perturbation of ensembles is critical to establishing a causal link between ensemble activity and behavior. Previous work used pattern completion properties to demonstrate the role of ensembles in visual perception: activation of ensembles via pattern completion neurons was sufficient to drive learned behavior in the absence of visual stimuli [8]. However, ensemble activation using the selected pattern completion neurons had a low success rate. To efficiently activate ensembles, neuroscientists need a way to optimize selection of pattern completion neurons. Proper design of perturbation patterns based on ensemble recordings could facilitate a more comprehensive understanding of ensemble function.

Recent advances in microscopy and protein engineering have partially developed the relationship between ensemble activity and behavior [18–25]. Advances in microelectrode arrays and fluorescence imaging have enabled neuroscientists to measure ensemble activity within and between cortical layers [10,26,27]. This work has shown that different ensembles are spatially overlapped and sequentially activated [8,28–31]. Given these properties, optogenetics has become valuable for ensemble manipulation due to its ability to selectively perturb individual neurons with temporal specificity [23,24,32,33]. Previous work has combined optogenetics and fluorescence imaging to study the spontaneous and evoked activation of ensembles [2,10]. This all-optical approach can facilitate the study of pattern completion and ensemble function *in vivo*.

Efficient selection of pattern completion neurons is important for analyzing ensemble dynamics and understanding the functional role of pattern completion in cognition. Previous work using conditional random fields to identify pattern completion neurons in layer 2/3 of mouse primary visual cortex (V1) resulted in an ensemble recall rate of 5% (i.e. 5% of trials successfully activated the ensemble) [8]. Despite some progress, there remains a limited understanding of the network properties that facilitate pattern completion.

Graph theory may be a useful tool to identify influential nodes in complex networks that drive pattern completion activity [34–40]. Graph theory applications in neuroscience have been effective, mainly in the context of understanding information transfer between brain regions or to understand network restructuring in disease. Examples of graph theory in these whole-brain cases have used either diffusion tensor imaging to extract structural connectivity or fMRI signals to estimate functional connectivity [41–45]. However, there is limited application of graph theory to analyze neural microcircuits, specifically cortical ensembles. Studying these microcircuits *in vivo* requires recording ensemble dynamics with high spatial, temporal, and genetic specificity. Neural relationships, such as correlations between spikes, help estimate network connectivity. Estimates of connectivity are limited by the slow temporal kinetics of calcium indicators, or the low spatial resolution and lack of genetic specificity of microelectrode arrays [46–50]. In comparison, a computational approach could analyze subthreshold dynamics and network connectivity while maintaining high spatial and temporal resolution.

In this study, we developed a computational model that simulated layer 2/3 of mouse V1. Computational models of layer 2/3 of V1 have been a useful tool to study circuit motifs that facilitate different activity patterns involved in visual processing [51–53]. Computational models have also been employed to investigate network responses to stimulation [54,55]. Recent work used a rate-based model of layer 2/3 of mouse V1 to analyze the network-wide effects of single neuron perturbation [55]. Their findings helped elucidate the circuit mechanism underlying feature suppression in excitatory cortical neurons. We built off these studies to develop a realistic spiking model of layer 2/3 of mouse V1 to observe and manipulate ensemble dynamics. Our model consisted of excitatory pyramidal neurons and three types of inhibitory neurons (Parvalbumin (PV), Somatostatin (SOM), and Vasoactive intestinal peptide (VIP) expressing neurons). Each neuron class in our model replicated connectivity patterns and functional properties found in slice and *in vivo*. We used K-means clustering to identify ensembles. We simulated optogenetic perturbation of neuron pairs within an ensemble to identify and characterize efficient pattern completion neurons over hundreds of trials. We then applied graph theory to predict efficient pattern completion neurons. Finally, we developed a novel metric to identify pattern completion neurons that could be experimentally acquired with modern recording techniques.

## Results

### Our model of V1 layer 2/3 replicated structural and physiological properties found in mice

**Fig 1:**
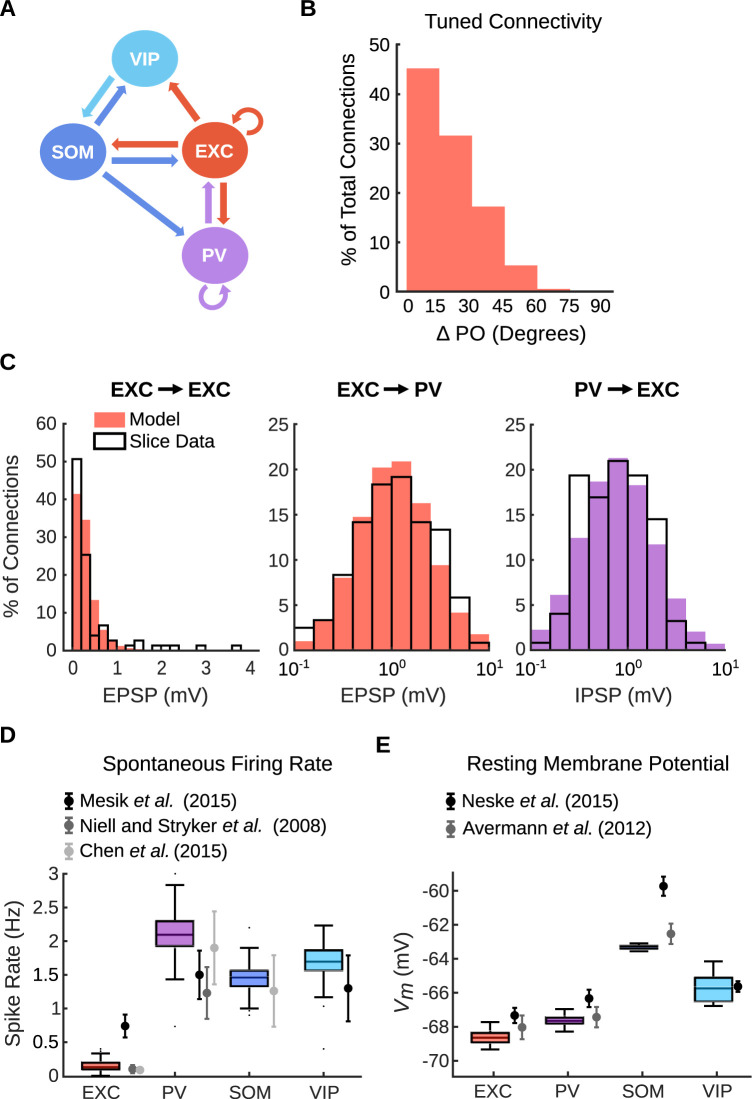
Model parameters are consistent with *in vivo* quantities. (A) Schematic of the connectivity pattern between excitatory neurons and inhibitory neurons in our model. (B) The probability of connection between excitatory neurons decreases as the difference between their PO increases. (C) Distribution of weights (represented as PSPs) between select neuron subtypes. Our model (filled bars) closely match the PSP distribution found by Cossell et al. [57] (*left*) and Znamenskiy et al. [60] (*middle/right*). (D) The average spontaneous firing rates in our model over 30 seconds resemble those found *in vivo* in anesthetized mice in Mesik et al. [63], Neill and Stryker [64], and Chen et al. [62]. Data points and error bars from *in vivo* data are the mean ± s.e.m. found in those studies. (E) Average resting membrane potential for each neuron subtype in our model over 30 seconds. Our results closely match those found *in vivo* in L2/3 of the barrel cortex in mice in Neske et al. [65] and Avermann et al. [66]. Data points and error bars from *in vivo* data are the mean ± s.e.m. found in those studies.

We attempted to accurately model the connectivity patterns and physiological properties found in layer 2/3 of the mouse visual cortex (*Methods*). First, the network mimicked the composition and connectivity pattern of the mouse cortex (Figs 1A and S1) [51–53,56,57]. The network comprised 4000 leaky integrate-and-fire neurons representing four different cell types. Excitatory pyramidal neurons made up 80% of the network, while inhibitory (PV, SOM, and VIP) neurons made up the remaining 20% of cells. Excitatory neurons made connections with other excitatory neurons and all inhibitory neuron subtypes, PV neurons connected to excitatory neurons and had recurrent connections with other PV neurons, and SOM neurons connected to all other neuron subtypes except themselves [51,53,56]. Neurons were assigned a preferred orientation (PO) between 0 and 180 degrees, which influenced connectivity between some cell types (*Methods*; S1 Fig) [51,55]. Excitatory neurons with similar POs were more likely to be connected to each other than to neurons with different POs (Fig 1B) [57–59]. The distribution of synaptic strengths between excitatory and PV cells was similar to distributions found in slice (Fig 1C) [57,60]. Strengths between all other cell types followed lognormal distributions with parameters guided by previously published studies in slice (S1B Fig) [56,61]. Second, the spiking statistics and baseline activity also matched experimentally obtained metrics. The spontaneous firing rate for each neuron subtype in the network resembled the corresponding firing rate found in anesthetized mice (Fig 1D) [62–64]. The baseline resting membrane potential for each neuron type closely resembled values found *in vivo*; SOM neurons had a higher baseline resting potential than the other neuron types (Fig 1E) [65,66]. The baseline membrane potential fluctuations were also in line with those found in whole-cell patch clamp measurements *in vivo* (S2A Fig) [67,68].

### Our model of V1 layer 2/3 recapitulated known responses to stimulation

**Fig 2:**
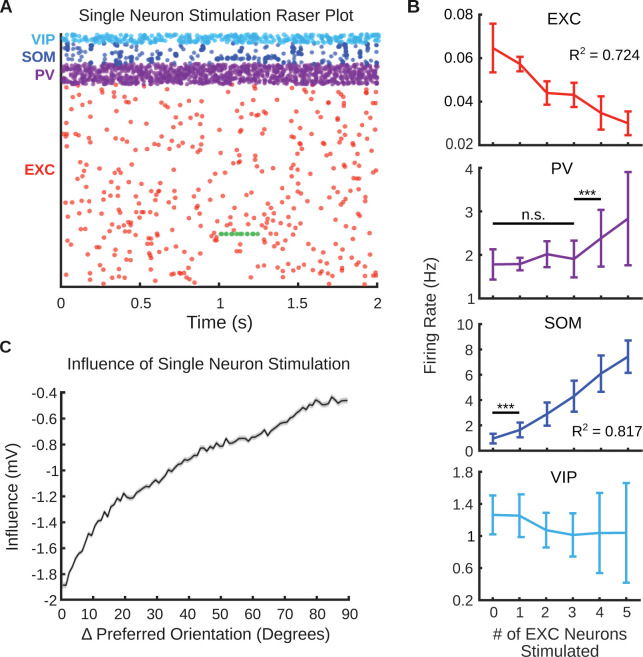
Model stimulation ensemble responses are concordant with *in vivo* findings. (A) Raster plot showing the firing patterns of the different neuron populations. Green dots represent evoked spikes from 30Hz stimulation of a single excitatory neuron for 250 ms. (B) Average firing rate of different neuron types during stimulation of 0 to 5 random excitatory neurons. Averages are over 20 trials (250 ms each). Error bars are mean ± standard deviation. *** indicates *p* < 0.001, n.s. indicates not significant. (C) Average change in membrane potential of excitatory neurons during stimulation of a single excitatory neuron. 35 neurons were individually stimulated for three 250 ms trials. The shaded region is the mean ± s.e.m of the change in membrane potential of all non-stimulated excitatory neurons in the network.

The network’s response to simulated optogenetic stimulation reproduced features of the response from *in vivo* mouse studies. We applied stimulation at 30 Hz for 250 ms duration (*Methods*). The stimulated neuron repeatedly fired during the stimulation period (Figs 2A and S2B). Single neuron stimulation produced a net inhibitory effect on the network (Fig 2B and 2C) [69]. The effect of stimulation was additive: increases in the number of stimulated excitatory neurons increased the inhibitory effect on the network (Fig 2B). Specifically, the average firing rate of excitatory neurons decreased linearly as the number of stimulated neurons increased (slope = −0.007 spikes/s/stimulation, *p =* 5.2 × 10^−35^, *F*-test, *n* = 20 trials for each stimulation number). The diverging properties and functions of neuron subtypes in the network matched experimental observations. SOM neurons in layer 2/3 responded to stimulation of a single excitatory neuron *in vivo* whereas PV neurons monitored population activity [70]. The average firing rate of SOM neurons increased linearly with the number of excitatory neurons stimulated (slope = 1.35 spikes/s/stimulation, *p* = 1.3 × 10^−45^, *F*-test, *n* = 20 trials for each stimulation number). However, PV neuron activity did not increase until at least 4 neurons were stimulated. Our network was also concordant with the observation of feature suppression in excitatory neurons in layer 2/3. Stimulated neurons suppressed neurons with similar POs more than neurons with dissimilar POs (Fig 2C; slope = 0.014 mV/degree, *p* = 8.1 × 10^−54^, *F*-test, *n* = 105 trials, 3 trials each for 35 different stimulated neurons) [55,69]. This feature suppression was observed experimentally through specific excitatory–inhibitory connectivity; PV neurons most strongly innervated excitatory neurons that strongly activated them and shared their PO [55,60,71].

### K-means clustering identified ensembles of densely connected neurons

**Fig 3:**
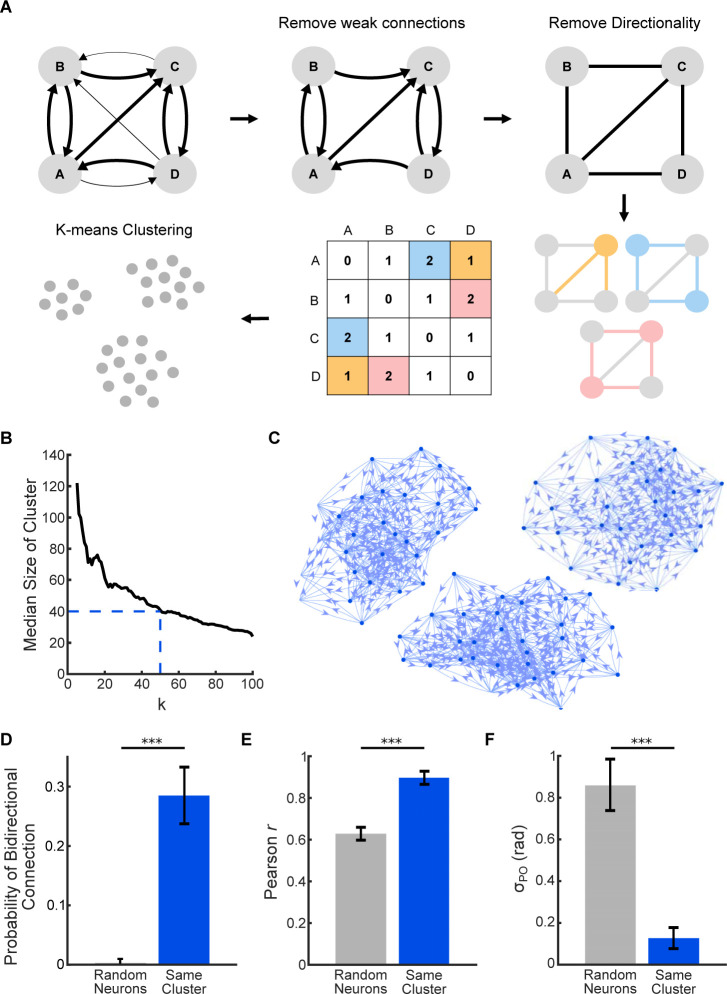
K-means clustering identified ensembles of densely connected neurons. (A) Steps to find ensembles of strongly connected neurons. *Top Left*: Example of network before preprocessing; *Top Middle*: We removed weak excitatory connections (EPSP < 0.9 mV) from the network. *Top Right*: The network was converted into an undirected graph. *Bottom*: After preprocessing, we computed a similarity matrix where each cell represented the number of shared excitatory connections between each pair of excitatory neurons. We then performed K-means clustering on this matrix to identify ensembles. (B) K-means clustering identified groups of densely connected neurons from the similarity matrix. The number of clusters was tuned such that the median size of each cluster (ensemble) was about 40 neurons (*dashed lines*). (C) Three examples of ensembles found in the network. We returned directionality (*arrows*) to the network after the ensembles were found. (D) Neurons within each ensemble had a higher probability of being bidirectionally connected than randomly selected neurons in the network. *n* = 10 sets of 100 neuron pairs. (E) Neurons within each ensemble were more correlated than randomly selected neurons in the network. *n* = 10 sets of 10 neurons. (F) Neurons within each ensemble had more similar POs than randomly selected neurons in the network. *n* = 10 sets of 10 neurons. *** indicates *p* < 0.001.

We used machine learning to identify clusters in the network that shared properties with neural ensembles found *in vivo*. Given the pattern completion properties of *in vivo* ensembles as well as their regular spontaneous activation, we reasoned that ensembles consisted of excitatory neurons with strong recurrent connections [2,8,12,72–74]. We constructed such ensembles by removing inhibitory neurons and weak connections from the network and then using K-means clustering to group densely connected excitatory neurons (*Methods*; Figs 3A, 3B and S3A). All but one of the fifty clusters showed orientation selectivity, and we did not classify the non-orientation selective cluster as an ensemble (S3B Fig). The resulting clusters were qualitatively densely interconnected (Fig 3C). Neurons within a cluster had a higher probability of being bidirectionally connected than randomly chosen neurons in the network (Fig 3D, *p =* 5.5 × 10^−5^, one-tailed Wilcoxon rank-sum test, *n* = 10 sets of 100 neuron pairs). To verify that the clusters also described the functional, non-structural properties of neural ensembles, we tested whether the neurons within an ensemble had more correlated activity and more similar POs than would be expected by chance. Voltage traces from neurons within an ensemble had higher correlation values than the correlation values between randomly selected neurons (Fig 3E, *p* = 9.1× 10^−5^, one-tailed Wilcoxon rank sum test, *n* = 10 sets of 10 neurons). The POs of neurons within an ensemble were also more similar than POs of randomly selected neurons (Fig 3F, *p* = 9.1× 10^−5^, one-tailed Wilcoxon rank sum test, *n* = 10 sets of 10 neurons), which is consistent with groups of these neurons responding to a “Go” stimulus of a certain orientation [8]. These findings were consistent across a range of median ensemble sizes (S4 Fig).

### A novel metric characterized the pattern completion capability of a pair of ensemble neurons

**Fig 4:**
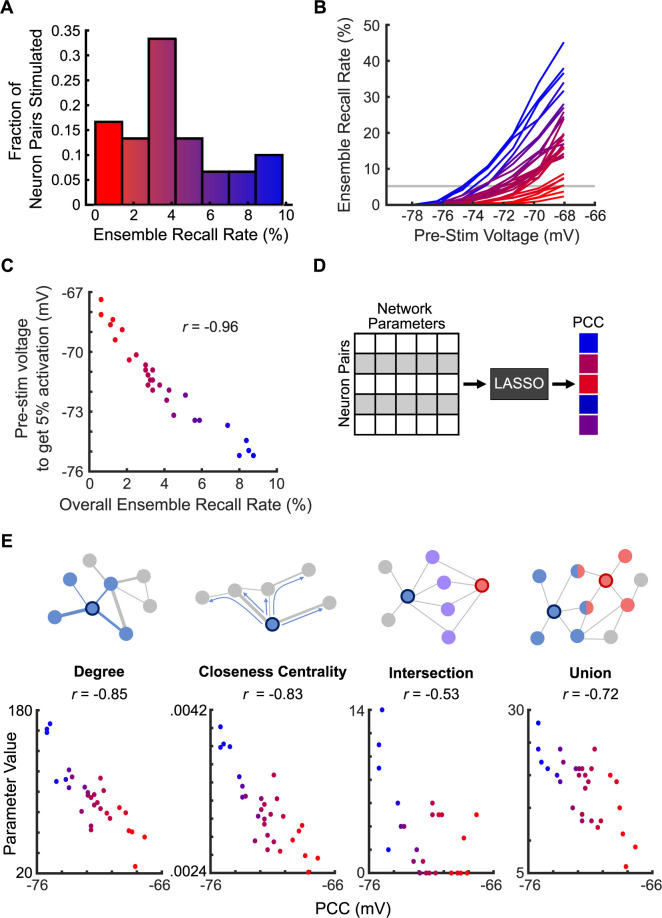
Pattern Completion Capability was correlated with various graph theory parameters. (A) Histogram of ensemble recall rate of 30 different pairs of neurons from ensemble 1. Neuron pairs with higher ensemble recall rates were better pattern completion neurons. Each pair was stimulated 800 times. The ensemble recall rate (the percent of trials where most of the ensemble was activated following stimulation) was calculated for each of the 30 pairs. (B) Probability of ensemble activation was dependent on the average membrane voltage of ensemble neurons before stimulation of the neuron pair. Neuron pairs with a higher overall ensemble recall rate were more likely to activate ensembles even at lower pre-stimulation voltages. (C) The pre-stimulation voltage needed for stimulation of a neuron pair to activate an ensemble 5% of the time was significantly and negatively correlated with the overall ensemble recall rate (*p* = 5.5 × 10^−16^, *F*-test, *n* = 29 neuron pairs). Neurons with higher overall ensemble recall rates could achieve a 5% recall rate at voltages further from threshold. (D) Graph theory parameters helped predict the pattern completion capability of each neuron pair through LASSO regression. (E) *Top*: Schematics representing the four network parameters calculated for each neuron pair. *Bottom*: The relationship between each network parameter and the pattern completion capability of each neuron pair. All variables were significantly and negatively correlated with PCC, with degree and closeness centrality having the strongest correlation (from left to right *p* = 4.6 × 10^−9^, 1.9 × 10^−8^, 3.3 × 10^−3^, 1.3 × 10^−5^, *F*-test, *n* = 29 neuron pairs).

We developed a metric to evaluate and compare the ensemble recall performance of different pattern completion neurons. We chose an ensemble at random to “train” by increasing the weights between ensemble neurons, simulating increased excitability found in ensembles (*Methods;* S5 and S6 Figs) [75]. We then randomly selected 30 different pairs of ensemble neurons to stimulate, matching experimental optogenetic perturbations [8]. We defined an ensemble as “activated” if 75% of ensemble neurons fired within 10 ms of stimulation. The average ensemble recall rate was just under 5%, which was consistent with *in vivo* experiments (Fig 4A) [8]. The average voltage of the ensemble just before stimulation was variable, and the probability of recalling an ensemble increased as the pre-stimulation voltage approached the −55 mV action potential threshold (Fig 4B). Some neuron pairs could successfully recall an ensemble at lower voltages, whereas other pairs only activated the ensemble when the ensemble was close to threshold. To account for variability in pre-stimulation voltage, we defined a metric that was independent of the distribution of pre-stimulation voltages for any given neuron pair. We termed this metric as the Pattern Completion Capability (PCC) and defined it as the pre-stimulation voltage needed for a neuron pair to achieve a 5% ensemble recall rate (*Methods*; S5C Fig). Neuron pairs with higher overall ensemble recall rates were able to achieve a 5% activation rate at pre-stimulation voltages further from threshold. PCC was highly negatively correlated with overall ensemble recall rate (Pearson *r* = −0.96) (Fig 4C).

### Graph theoretic connectivity parameters of neurons predicted neuron PCC

**Fig 5:**
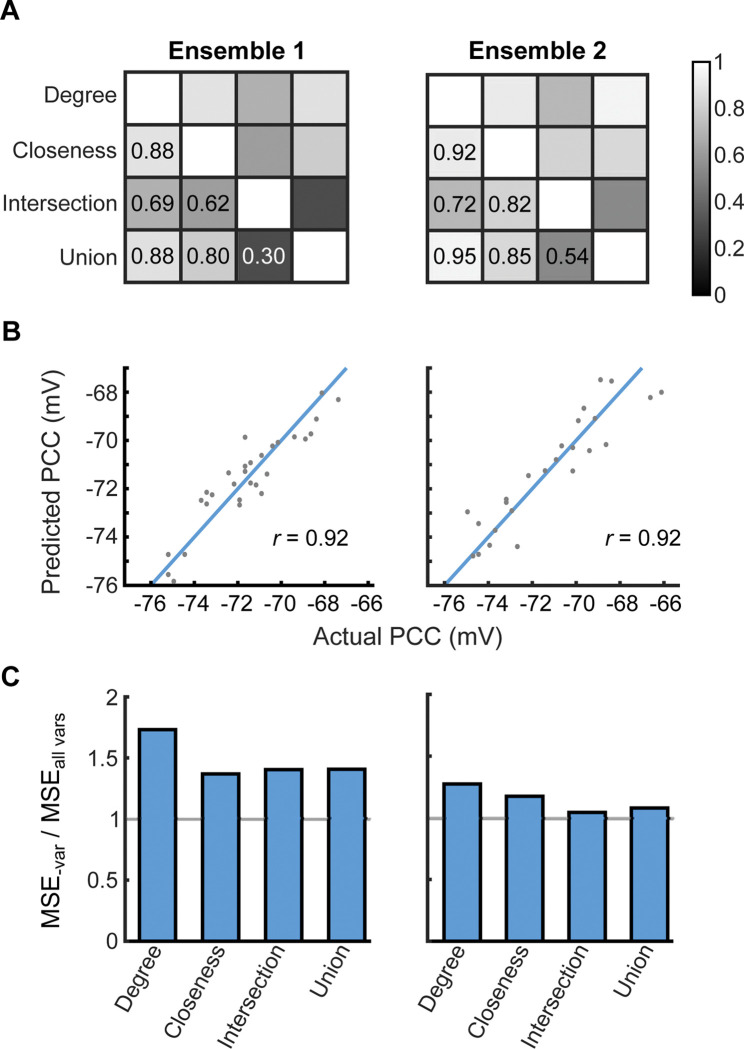
LASSO regression can predict Pattern Completion Capability. (A) Correlation matrix of the network parameters for each of the 30 neuron pairs in ensemble 1 (*left*) and each of the 25 neuron pairs in ensemble 2 (*right*). (B) LASSO regression accurately predicted the PCC of each neuron pair in ensemble 1 (*left*) and ensemble 2 (*right*). Actual PCC was calculated from 800 stimulation trials for each neuron pair. Separate LASSO models were computed for each ensemble. Blue line indicates *y* = *x*. (C) Algorithm reliance for each network parameter. For both ensemble 1 (*left*) and ensemble 2 (*right*), the accuracy of LASSO regression was most reliant on degree.

We were able to predict a neuron pair’s PCC from graph theory parameters (*Methods*). We used LASSO regression to account for multicollinearity that would arise between these parameters (Fig 4D). We used best subset selection to choose four graph theory parameters to use as predictors in our LASSO model: degree, closeness centrality, intersection, and union (*Methods*; Figs 4E, *top* and S7). Each of these measures was linearly related to PCC with varying strengths (Fig 4E, bottom). Degree and closeness centrality had the strongest relationship with PCC, followed by union and then intersection. Higher values for all parameters were associated with PCCs further from threshold, indicating higher efficiency at activating the ensemble. These parameters were also correlated with each other to varying degrees (Fig 5A).

LASSO regression accurately predicted PCC from the four network parameters (Pearson *r* = 0.92) (Fig 5B and S1 Table). We tested the importance of each parameter with respect to the model’s prediction accuracy by computing each parameter’s algorithm reliance value (*Methods*; Fig 5C). Model accuracy was most reliant on degree: removing degree from training resulted in a 1.7-fold increase in error. Additionally, removal of any of the variables from the algorithm increased the amount of error in predicted PCC, as indicated by algorithm reliance values greater than 1.

To test for reproducibility, we chose another ensemble at random, stimulated 25 random neuron pairs from that ensemble, and calculated the same network parameters for those pairs (S8 Fig). Trends in multicollinearity were similar between both ensembles (Fig 5A). LASSO regression accurately predicted PCC in this ensemble as well (Pearson *r* = 0.92) (Fig 5B). Model accuracy was most dependent on degree, albeit to a lesser extent (algorithm reliance = 1.3) indicating that the other graph theory parameters were able to compensate for the loss of degree information.

### A novel latency metric helped identify pattern completion neurons

We next identified an alternative approach to identifying pattern completion neurons that accounted for the limitations of *in vivo* recordings. Although optical and protein engineering advancements have improved the temporal resolution and field of view of imaging setups, it remains difficult to reconstruct network connectivity using *in vivo* measurements. Calcium indicators do not measure sub-threshold voltages, which limits their ability to estimate EPSP and IPSP values. Voltage indicators typically have small responses and likewise cannot accurately quantify the postsynaptic strengths [76]. These challenges could hinder the assessment of the weights and directionality of connections *in vivo*.

**Fig 6:**
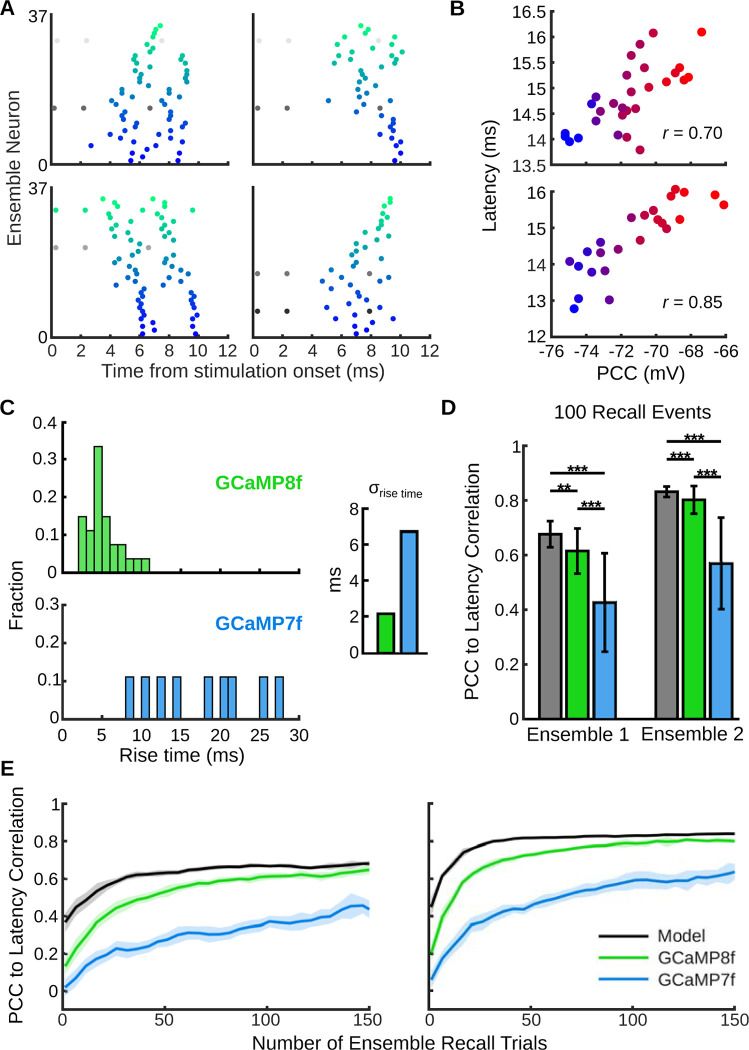
Modern GCaMP sensors may be able to identify efficient pattern completion neurons *in vivo*. (A) Raster plots of four example ensemble recall events (from ensemble 1) evoked by stimulation of different pairs of neurons. Neurons in the ensemble activated in a sequential manner rather than simultaneously. The *y*-axis spans all neurons in the ensemble. (B) Scatter plot of average latency vs. PCC in ensemble 1 (*top*) and ensemble 2 (*bottom*) for all 800 trials of each neuron pair. (C) Distribution of 0–80% rise time for GCaMP8f and GCaMP7f. Data were taken from previous research that performed simultaneous calcium imaging and cell attached electrophysiology in mouse visual cortex [81]. *Inset*: The standard deviation of GCaMP8f’s rise time was less than that of GCaMP7f. (D) Effect of calcium indicators on Pearson *r* between latency and PCC when calculated with 100 trials. Error bars represent mean ± standard deviation. ** indicates *p* < 0.01 and *** indicates *p* < 0.001. (E) Accuracy of latency measurement as a function of the number of ensemble recall events for ensemble 1 (*left*) and ensemble 2 (*right*). The correlation between latency and PCC increased as the number of ensemble recall events increased and as the temporal dynamics of calcium indicators became less variable. The shaded region is the mean ± s.e.m of the correlation coefficient.

However, the improved kinetics of modern calcium and voltage indicators can obtain useful information about ensemble dynamics. Our method of estimating PCC *in vivo* can take advantage of the sequential activation pattern of ensembles; neurons activate in stages rather than simultaneously (Fig 6A) [28–30,77–80]. This pattern of activation was not uniform over our trials. We hypothesized that, on average, neurons that fired earlier in the sequence would have better pattern completion abilities than neurons that fired later. We labeled this metric “latency,” and found that it was highly correlated with PCC in both ensembles (Pearson *r* = 0.70 and 0.85) (*Methods*; Fig 6B).

The variable rise kinetics of popular calcium indicators can hinder estimation of spike timing [48]. We next evaluated how variability of *in vivo* optical imaging conditions, specifically those of modern GCaMP variants, would affect the measurement of latency (*Methods*) [19,81]. The rise time variability of GCaMP8f is less than that of GCaMP7f (Fig 6C). Variability of these sensors impaired measurement of latency as shown by a weakened correlation between PCC and latency in both ensembles (Fig 6D; *p*-values in S2 Table, one-tailed Wilcoxon rank-sum test, *n* = 50 sets of 100 ensemble activation events).

We next examined the number of ensemble recall events required to predict PCC using different calcium indicators. For both ensembles, GCaMP8f was able to achieve a Pearson *r* between latency and PCC greater than 0.5 in under 50 events (Fig 6E). However, GCaMP7f did not reach a Pearson *r* of 0.5 in under 150 events in ensemble 1 and needed 65 events in ensemble 2. Taken together, these results suggest that GCaMP8f could be used *in vivo* to identify pattern completion neurons from a reasonable number of ensemble activation events.

### Latency from spontaneous ensembles identified pattern completion neurons

**Fig 7:**
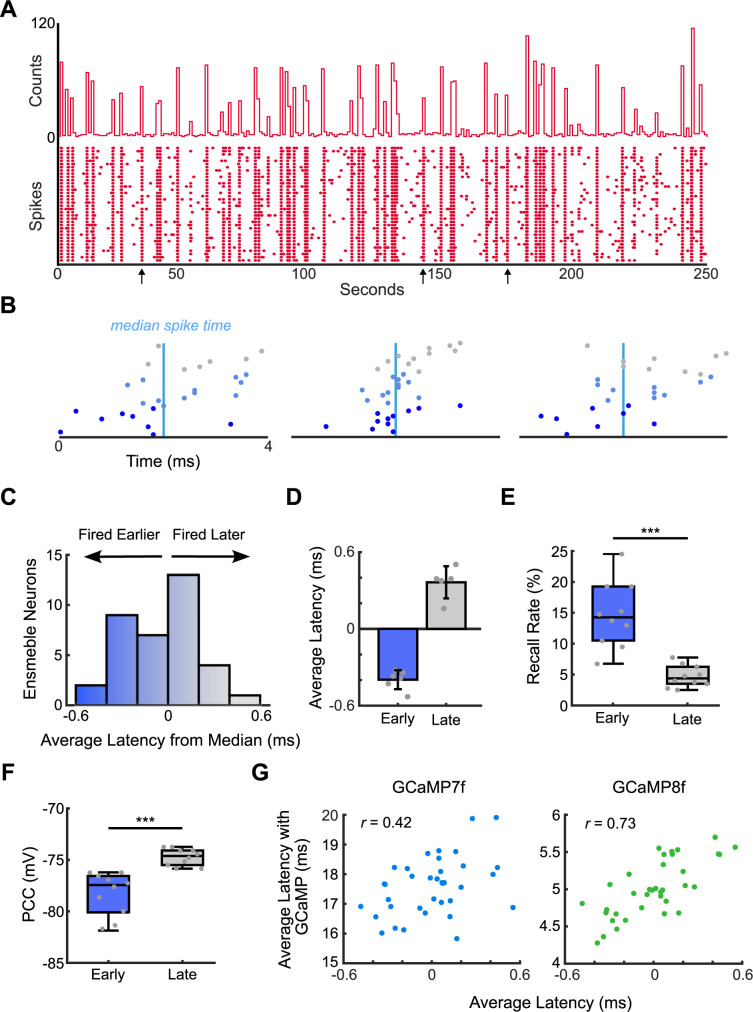
Latency within ensemble activation events during spontaneous ensemble activity can identify pattern completion neurons. (A) *Top*: Histogram of the number of spikes from 36 ensemble neurons over 250 seconds (using 1 second bins). *Bottom*: Raster plot of spontaneous ensemble activity from all 36 ensemble neurons. Arrows represent ensemble events shown in (B). (B) Zoomed-in view of three ensemble recall events. The cyan vertical line represents the median spike time, which was the reference for calculating relative latency for that recall event. Neurons are arranged and colored by latency, with earlier average latency neurons closer to the *x*-axis. (C) Histogram of the distribution of latency for each ensemble neuron. Latency was averaged over 41 ensemble recall events. The color gradient of the bars also serves as the colormap for individual neurons in panel (B). (D) 6 neurons that had early (negative) average latency values and 5 neurons that had late (positive) average latency values were selected to stimulate in pairs. (E) Early neuron pairs had a higher ensemble recall rate over 400 stimulation events than late neuron pairs. *n* = 10 neuron pairs for each group. (F) Early neuron pairs had a lower PCC than late neuron pairs. *n* = 10 neuron pairs for each group. (G) A scatter plot for the two forms of latency demonstrates that latency calculated from the action potential model was generally correlated with the latency calculated from the model incorporating GCaMP rise kinetics for all ensemble neurons from 41 spontaneous recall events. *** indicates *p*<0.001.

Next, we tested our latency metric on spontaneous ensemble recall events. Ensembles *in vivo* have regular spontaneous activity, and we hypothesized that neurons that fired earlier in spontaneous ensemble events would be better pattern completion neurons [4]. We selected a new ensemble (*n* = 36 neurons) and observed spontaneous activity for 250 seconds (Fig 7A). We detected 41 spontaneous ensemble recall events during this period. We calculated latency as the relative time between each neuron spike and the median time for each ensemble event (*Methods*; Fig 7B). We averaged the latency for each neuron over the 41 events (Fig 7C). We identified 6 neurons with early average latency and 5 neurons with late average latency (Fig 7D). We randomly defined 10 pairs of early neurons and 10 pairs of late neurons drawn from our defined neurons without replacement, stimulated each pair 400 times, and calculated the recall rate and PCC for each pair. Neurons with earlier latency during spontaneous ensemble events were better pattern completion neurons than neurons with higher latency. Neuron pairs that, on average, fired early in spontaneous recall events had significantly higher recall rates during paired stimulation (Fig 7E; *p* = 1.4 × 10^−4^, one-tailed Wilcoxon rank sum test, *n* = 10 pairs of neurons). Early neurons also had significantly lower PCC than later neurons (Fig 7F; *p* = 8.9 × 10^−5^, one-tailed Wilcoxon rank sum test, *n* = 10 pairs of neurons). Finally, we found that simulated action potential latency was correlated with calcium latency when simulated using experimental GCaMP rise kinetics (Fig 7G).

### Stimulation of five neurons could reliably activate ensembles

**Fig 8:**
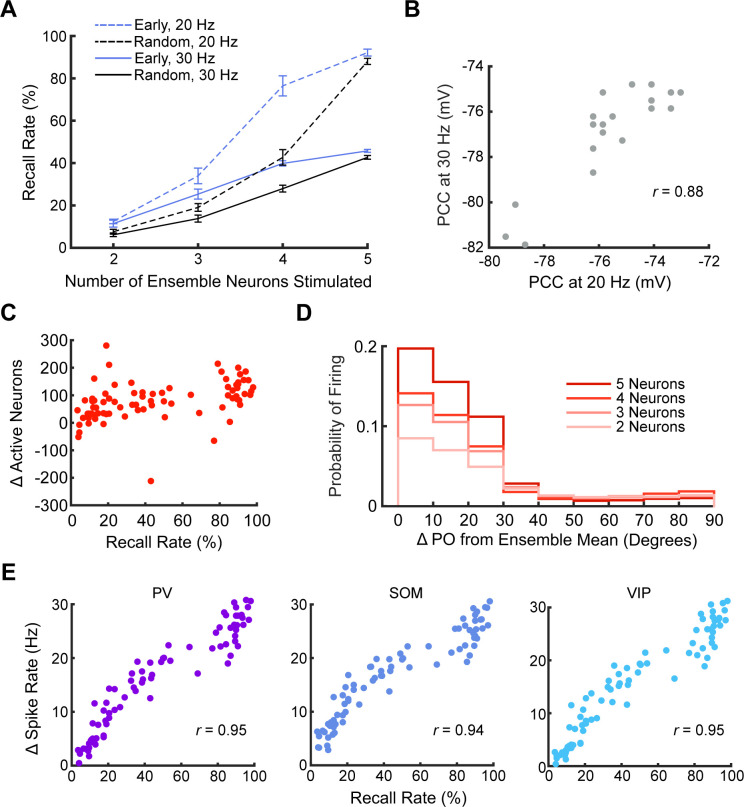
Stimulation of five neurons can reliably activate ensemble neurons. (A) Plot of the number of neurons stimulated vs. average ensemble recall rate for each stimulation condition, varying neuron selection and stimulation frequency. Error bars represent standard error (*n* = 10 neuron groups at each stimulation group size). (B) PCC from the same neuron pairs stimulated at both 20 Hz and 30 Hz (*n* = 20 neuron pairs). PCC from the two stimulation frequencies were well correlated. (C) The change (relative to baseline) in the number of non-ensemble excitatory neurons that fired during the stimulation period vs. ensemble recall rate. Recall rate was increased by increasing the number of stimulated ensemble neurons (*n* = 80 neuron groups of size 2 to 5). (D) Histogram of the probability that a non-ensemble excitatory neuron spiked as a function of their PO relative to the ensemble’s mean PO. Similar POs became more likely to spike during the stimulation period as ensemble recall rate increased (measured by number of early neurons stimulated) (*n* = 40 neuron groups of size 2 to 5 stimulated for fifty 400 ms periods). (E) Change in average spike rate (relative to baseline) for each inhibitory neuron group as a function of ensemble recall rate (*n* = 80 stimulation periods).

Finally, we evaluated the number of stimulated neurons needed to reliably activate ensembles. Stimulation of two pattern completion neurons could achieve a median recall rate of about 15% (Fig 7E). Using the same ensemble as in Fig 7, we identified the seven neurons with the earliest latency and randomly sampled 10 sets of 2 to 5 of these neurons without replacement. We also randomly sampled sets of neurons with varying latency from the entire ensemble. We stimulated each of these neuron sets 200 times at both 20 Hz and 30 Hz (Fig 8A). Randomly sampled neurons consistently had a lower recall rate than early neurons. We found that 20 Hz stimulation of either four early neurons or five random neurons could reliably activate ensembles (recall rate >75%). 30 Hz stimulation did not allow enough time between trials for the ensemble to recover from the previous recall event (S9 Fig). Importantly, PCC calculated from the same neuron pairs stimulated at both 20 Hz and 30 Hz were consistent, indicating that PCC was a robust measure of a neuron pair’s performance (Fig 8B; Pearson *r* = 0.88, *n* = 10 pairs of early neurons and 10 random pairs of neurons). We then analyzed the response of the rest of the network when the ensemble was stimulated at 20 Hz for 400 ms. As the recall rate increased, the number of active excitatory neurons increased compared to pre-stimulation (Fig 8C). The neurons that were active during this period were more likely to have similar POs to ensemble neurons (Fig 8D). The probability of these neurons firing during the stimulation period increased as the recall rate/number of stimulated neurons increased. These results were consistent with those found *in vivo* [10]. To balance this increased excitatory activity, each group of inhibitory neurons increased their firing rate during the stimulation period compared to baseline (Fig 8E). This increase was directly correlated to recall rate for each inhibitory neuron group (*n* = 80 neuron groups, sizes 2 to 5).

## Discussion

In this work, we developed a spiking model of layer 2/3 of mouse V1 to evaluate properties of pattern completion neurons in cortical ensembles. Our model replicated known connectivity patterns and functional properties found in slice and *in vivo*. Our model consisted of 80% excitatory neurons, and the remaining 20% of neurons was split into three interneuron classes: PV, SOM, and VIP neurons. Synaptic weights between these cell types were log-normally distributed and these distributions aligned with postsynaptic potentials found in slice. Connections were governed by PO similarity, which inherently created ensembles. Additionally, fundamental physiological properties of each neuron class, such as firing rate and resting membrane potential, replicated values found *in vivo*. The functional properties of specific neuron classes in our model were also concordant with *in vivo* findings. Excitatory neurons in our model exhibited feature suppression; SOM neurons monitored single neuron activity while PV neurons monitored population activity. Finally, ensembles in our model exhibited characteristics found *in vivo*: ensembles displayed pattern completion properties and activated sequentially.

We then developed methods to optimize selection of pattern completion neurons. We defined a novel metric, PCC, that described the pre-stimulation voltage needed for pattern completion neurons to have a 5% success rate. Efficient pattern completion neurons were able to activate ensembles at lower pre-stimulation voltages. We found that LASSO regression could accurately predict PCC using graph theory parameters. Of these parameters, degree had the strongest correlation with PCC. We next defined a novel metric, latency, that could be used in future calcium imaging studies to identify pattern completion neurons *in vivo*. Latency could be used to predict PCC in both evoked and spontaneous ensemble events. Finally, we found that stimulation of five ensemble neurons could reliably activate ensembles.

Our computational approach allowed us to identify and characterize network properties that optimize ensemble recall. Previous work has used graph theory to identify pattern completion neurons *in vivo* [8,82]. These studies used conditional random fields (CRFs) to identify neurons that predicted features of visual stimuli associated with their ensemble. When tested with optogenetic stimulation *in vivo*, these pattern completion neurons had a 5% ensemble recall rate. Pattern completion neurons in our model also had an average recall rate of 5%. Our computational approach improved our understanding of network connectivity and enabled simulation of subthreshold dynamics. We found that ensemble neurons had highly correlated membrane potentials, and ensemble recall probability was dependent on the average membrane potential of the ensemble just before stimulation. We then applied graph theory and machine learning to identify network properties that optimized ensemble recall rate.

The relationship between network architecture and driver nodes has varied across studies in multiple disciplines. Multiple methods have been proposed to identify influential nodes in theoretical complex networks [40,83–86]. Network-based methods have also been used to identify influential nodes in a variety of real networks including metabolic, cell-signaling, and social networks [87–90]. Theoretical methods have generally found that centrality measures such as betweenness centrality are better predictors of influence than degree. However, the properties of optimal control nodes varied with network type; driver nodes in theoretical networks typically had low degree while driver nodes in biological networks had higher degree values [88,91–94]. Neuroscience networks likely also have unique properties that affect node influence.

Our analysis of network theory focused on a regime of network theory underexplored by previous studies. Specifically, neural networks are usually directed, spiking networks with both excitatory and inhibitory connections. These networks also have complex subthreshold dynamics that vary over time. Our work expanded on previous computational and systems neuroscience studies to apply graph theory to cortical ensembles. The growing field of network neuroscience aims to identify influential nodes in structural and functional brain networks using network theory and control theory [37,39,43,45,95,96]. However, nodes in these networks usually refer to brain regions rather than individual neurons [35,41,44,97–99]. Our work focused on identifying pattern completion neurons in cortical microcircuits. Moreover, our work focused on finding an optimal *pair* of influential nodes, rather than a single influential node. Nevertheless, our finding that the degree of these neuron pairs strongly predicted influence on the rest of the network matched similar observations in other networks [93].

Future work that follows from our simulated results could validate and apply our findings *in vivo*. Researchers can estimate graph theory parameters by reconstructing network connectivity using electrical or optical modalities. Network connectivity has been estimated by calculating the cross-correlation, transfer entropy, or Pearson *r* between electrical or fluorescence traces from individual neurons [46,100–107]. Multiple recording modalities can obtain traces from ensemble neurons *in vivo*, each of which has distinct advantages and disadvantages. First, microelectrode arrays can be used to study cortical dynamics with high temporal precision [50,108–111]. However, these electrodes record extracellular field potentials from multiple surrounding neurons and therefore lack the spatial and genetic specificity offered by genetically-encoded fluorescent indicators. Genetically-encoded calcium indicators provide high spatial resolution and can be genetically targeted to study specific cell types. However, the temporal dynamics of these sensors are slower and more variable than electrode measurements [19,47,48,112,113]. This slow temporal resolution may limit functional connectivity estimation to undirected networks. However, the amplitude and temporal kinetics of these indicators have iteratively improved over the past decade; the signal-to-noise and temporal resolution of GCaMP8f are 5 times better than those of GCaMP6f [19,113]. Recent advances in protein engineering have designed genetically-encoded voltage indicators that can record membrane potentials with genetic specificity and high spatial and temporal resolution [76,114–117]. However, capturing millisecond temporal dynamics requires an imaging frame rate of at least 500 Hz. Additionally, the signal-to-noise ratio of these indicators is an order of magnitude worse than that of calcium indicators. For these reasons, voltage imaging is not currently feasible for neural population studies in most lab settings. The further development of calcium indicators as well as the emergence of voltage imaging will likely improve the estimation of network connectivity. Improved temporal precision of calcium indicators will help estimate direction of connectivity, while analysis of subthreshold dynamics via voltage imaging will improve estimation of connection strength [76,113,115–118]. Nevertheless, our investigation of the network parameters that predict pattern completion ability could potentially inform macro-scale studies, where network connectivity between brain regions is achievable. Such information could be used to study conditions such as seizures, which have been shown to exhibit stereotypical sequential activations of different brain regions, similar to ensemble activation events [119–121]. However, for studies at the micro-scale *in vivo*, we recommend researchers use our latency metric or stimulate at least five ensemble neurons.

To account for limitations in network reconstruction, we defined a novel latency metric that predicted pattern completion neurons *in vivo* that was independent of network parameters. Our latency metric required repeated activation of ensembles, which can be achieved using holographic optogenetics or the repeated presentation of sensory stimuli [4,8,10,18]. We found that a robust relationship between latency and PCC was achieved in less than 100 trials using GCaMP8f. This aligned with the number of trials used in a single session to train mice on a visual discrimination task [122,123]. The entire process of identifying an ensemble, activating it tens to hundreds of times, and then calculating latency, can be accomplished in a few hours. Pattern completion experiments can then be performed directly after or on subsequent days, as imprinted and visually-evoked ensembles are stable across days [1,2]. However, given the temporal precision needed to accurately measure latency, experimentalists using this technique should use imaging setups that can achieve fast (>200 Hz) frame rates [124–126].

A computational model can interrogate network response to repeated activations of ensembles in a fraction of the time of experimental protocols but has inherent limitations. It is possible that pattern completion properties of ensembles appear only after training by repeated co-activation of a subset of ensemble neurons [10,11,127,128]. Recent work suggested an “Iceberg Model” of ensemble activation where neuronal excitability increases during the training of ensembles [75]. In support of this theory, connections between ensemble neurons were found to have a lower action potential threshold in slice and these neurons also exhibited increased membrane resistance after training. To simulate increased neural excitability, we increased the weights of connections between ensemble neurons during ensemble stimulation trials. However, future computational models may consider incorporating alternative mechanisms to simulate increases in neural excitability. While our synaptic weights were static, plasticity mechanisms to change synaptic weights during training can be added to study neural ensemble formation and activation [129,130]. Additionally, researchers may manually alter the spike threshold and lower membrane resistance in ensemble neurons to replicate recent findings in slice [75].

In this study, we used K-means clustering to identify potential ensembles that we could then “train” by increasing the weights of connections between these neurons. A limitation of this method is that K-means clustering cannot group a neuron into more than one ensemble, while previous work has established that individual neurons can participate in multiple ensembles [4]. Another limitation of our method is that we trained a single ensemble at a time when many ensembles exist simultaneously. Finally, K-means clustering in our model relied on knowledge of network connectivity, which is difficult to obtain from imaging experiments. Thus, we suggest experimentalists identify ensembles by observing spontaneous or visually evoked activity *in vivo*.

Overall, our work can help researchers efficiently identify pattern completion neurons to methodically manipulate ensembles with precise temporal resolution. These experiments are critical to elucidating the functional roles that ensembles and pattern completion play in perception, memory, and behavior.

## Materials and methods

### Network connectivity

Our model consisted of 4000 neurons. We divided these neurons into four groups: excitatory neurons (3200 neurons), PV neurons (330), SOM neurons (330), and VIP neurons (140). We assigned each neuron a preferred orientation between 0 and 180 degrees, which determined the probability of connection between some cell types and the cell-to-cell connection strength (S1A Fig). The probability of connection between excitatory neurons was high if the neuron’s POs was parallel; neurons with perpendicular POs were least likely to be connected. The synaptic connection weight of a projection from neuron *i* to neuron *j*, *W*_*ij*_, was also proportional to PO similarity: the change in conductance that neuron *i* evoked in neuron *j* was greater between neurons with more parallel POs. *W* values between cell types followed a log-normal distribution. The mean and deviation of these connections was guided by their respective experimental values found in slice from previous studies (S1 Fig) [56,57,60].

### Neuron model

We used a leaky integrate-and-fire model to simulate voltage dynamics. We modeled synapses with a conductance in the post-synaptic neuron that changed with activity in a pre-synaptic neuron [130]. The sub-threshold membrane voltage of neuron *i*, *V*_*i*_, obeyed the equation:

$$\frac{dV_{i}}{dt}=\frac{1}{C_{m}}[-g_{L}(V_{i}-V_{L})-g_{i,E}(V_{i}-V_{E})-g_{i,I}(V_{i}-V_{I})+g_{opsin}(V_{i}-V_{opsin})+B].$$

The membrane capacitance was *C*_*m*_ = 200 pF and the leak conductance was *g*_*L*_ = 10 nS. Reversal potentials for excitatory and inhibitory channels were *V*_*E*_ = 0 mV and *V*_*I*_ = −80 mV, respectively. We tuned the leak reversal potential, *V*_*L*_, for each cell type to match the simulated resting membrane voltage to the experimental values; this resulted in *V*_*L*_ = −50 mV, −55 mV, −65 mV, and −65 mV for EXC, PV, SOM, and VIP neurons, respectively. Following an action potential, neurons experienced a refractory period of 2 ms.

The background current was *B* = 100 pA for excitatory neurons and 0 pA for inhibitory neurons. We simulated membrane voltage fluctuations with a noise amplitude of 5 mV obtained from *in vivo* patch clamp recordings [67,68].

We simulated optogenetic stimulation with a 250 ms train of 0.6 ms width pulses at 30 Hz. These pulses activated the optogenetic conductance *g*_opsin_ = 150 nS. The reversal potential for the simulated light-gated channel was *V*_opsin_ = 0 mV, based on the reversal potential for Channelrhodopsin 2 [131]. The rhodopsin was inactive (*g*_opsin_ = 0 nS) during periods without stimulation.

An action potential event in an excitatory neuron, *i*, increased the excitatory conductance of postsynaptic neuron *j*, *g*_*j*,*E*_, by the synaptic connectivity strength, *W*_*ij*_. Postsynaptic increases in conductance were delayed 1.5 ms from the presynaptic spike to account for conduction time and synaptic delay [132]. An action potential in an inhibitory neuron increased inhibitory conductance of postsynaptic neuron *j*, *g*_*j*,*I*_, by *W*_*ij*_. Postsynaptic increases in inhibitory conductance occurred between 4 and 6 ms after the presynaptic spike [133]. Spontaneous firing was simulated with Poisson input that increased *g*_*i*,*E*_ by 30 nS at different rates for each cell type (EXC = 0.08 Hz, PV = 1.6 Hz, SOM = 0.05 Hz, VIP = 1.3 Hz). Otherwise, these parameters followed dynamics governed by the equations:

$$\frac{dg_{i,E}}{dt}=\frac{-g_{i,E}}{\tau_{E}},\tau_{E}=5\;ms;\frac{dg_{i,I}}{dt}=\frac{-g_{i,I}}{\tau_{I}},\tau_{I}=10\;ms$$

The integration time step for our simulations was 0.1 ms. Simulations were programmed in Python using Brian2.0.

### Identifying ensembles

We first removed the weakest 80% of EXC-EXC connections from the network (EPSP < 0.9 mV). We then removed directionality from the network such that any neurons with either unidirectional or bidirectional synapses were considered connected. We created an adjacency matrix, *A*, where each cell *A*_*ij*_ represented the number of neurons that neuron *i* and neuron *j* were both connected to. We performed K-means clustering on *A* and tuned the number of clusters *k* until the median cluster/ensemble size was 40 neurons (S3A Fig). This neuron number was based on previous imaging experiments and assumed that those experiments underestimated ensemble size due to limitations in field of view and viral expression [1,10]. The median ensemble size did not affect correlation between ensemble neurons or deviation in PO for ensemble neurons (S4A and S4B Fig). Ensemble size had only a moderate effect on the probability of bidirectional connection (S4C Fig). However, in all tested ensemble sizes, the probability of bidirectional connection was greater between cluster neurons than between random neurons. In this study, we focused on orientation-selective ensembles; we randomly selected ensembles from the result of K-means clustering that had a PO standard deviation less than 0.25 radians (S3B Fig). Ensemble 1 in this paper had 37 neurons and ensembles 2 and 3 had 36 neurons. Directionality was added back to the network for further analyses.

### Stimulating pairs of neurons to observe ensemble response

We simultaneously stimulated pairs of neurons randomly chosen from an ensemble (30 pairs for ensemble 1 and 25 pairs for ensemble 2). Our simulations were eight 0.6 ms pulses delivered at 30 Hz. For subsequent 20 Hz stimulation, we delivered eight 0.6 ms pulses at 20 Hz. Each pulse successfully activated the ensemble if at least 75% of the neurons in the ensemble fired within 10 ms of the start of the pulse.

We altered the weights of connections between ensemble neurons to achieve a 5% ensemble activation rate by titrating the fold-increase in connection weight (*Discussion*, S5B Fig). We then altered the background current to *B* = 75 pA for excitatory neurons *to* maintain the baseline resting membrane potential and spontaneous firing rates found *in vivo* (S6 Fig).

### Pattern completion capability

We define pattern completion capability (PCC) as the mean membrane voltage of an ensemble of neurons in the immediate 10 ms before stimulation that corresponded with a 5% ensemble recall rate. We calculated this value by first calculating the pre-stimulation voltage of the ensemble, *V*_*e*_, as the average of the membrane voltage of the ensemble in the 10 ms leading up to stimulation for each trial. We then binned trials by their pre-stimulation voltage in a sliding window with a width of 5 mV and a step size of 1.6 mV. Because there were few trials at high mean ensemble voltages (S5A Fig), we excluded trials with mean voltage above −68 mV from analysis. We fit the recall rate to a logistic form:

$$Recall\;rate(V_{e})=\frac{1}{1+e^{-a(V_{e}-b)}},$$

where parameters *a* and *b* were fitting parameters found with nonlinear least squares optimization.

We achieved a strong fit for 29 of the 30 neuron pairs in ensemble 1 and all of the neuron pairs in ensemble 2 (*R*^2^ = 0.99 ± 0.12 for ensemble 1 and *R*^2^ = 0.98 ± 0.03 for ensemble 2) (S5C and S5D Fig). The pair in ensemble 1 with a weak *R*^2^ value was excluded from further analyses. We estimated PCC from each neuron pair’s fitted equation by setting recall rate equal to 5% and solving for *V*_*e*_.

### Graph theory parameters

Graph theory parameters were calculated from the network that removed weak connections (EPSP < 0.9 mV). We also removed all inhibitory neurons and all excitatory neurons outside of the ensemble from the network as well. Parameters were calculated as follows for a pair of neurons *A* and *B*:

- *Degree*: Weighted sum of all outgoing connections from Neuron *A* to other nodes in the ensemble plus the weighted sum of all outgoing connections from Neuron *B* to other nodes in the ensemble.
- *Closeness Centrality*: Inverse sum of the distance from Neuron *A* to all other neurons in the ensemble plus the inverse sum of the distance from Neuron *B* to all other neurons in the ensemble. Distance was quantified using the synaptic weight.
- *Intersection*: Number of neurons postsynaptic to both Neuron *A* and Neuron *B*.
- *Union*: Number of neurons postsynaptic to Neuron *A* plus the number of neurons postsynaptic to Neuron *B* minus the neurons postsynaptic to both Neurons *A* and *B*.

To select these parameters, we performed best subset selection on seven common network parameters and found that the model with these four parameters maximized accuracy and parsimony (S7 Fig). Parameters were calculated using MATLAB’s centrality function.

### LASSO regression

We used LASSO regression to predict PCC from graph theory parameters. LASSO regression uses *L*_1_ regularization to minimize the model coefficients, $\hat{\beta }$, while minimizing mean squared error across the ensemble neurons:

$${\hat{\beta }}_{LASSO}=\underset{\beta }{\mathrm{argmin}}\left(\sum_{i=1}^{n}({PCC}_{i}-x_{i}^{T}\;\hat{\beta }{)}^{2}+\lambda \sum_{j=1}^{4}\left|{\hat{\beta }}_{j}\right|\right)$$

In this model, ***x*_*i*_**, represented the 4 × 1 vector of graph theory parameters for the *i*^th^ neuron pair. PCC_*i*_ represented the PCC value for the *i*^th^ neuron pair. Thus, $x_{i}^{T}\hat{\beta }$ represented the predicted PCC value. We used leave-one-out cross validation to tune the penalty term, *λ*, for each ensemble.

### Algorithm reliance

We calculated algorithm reliance for each variable by removing that variable from training, training a new LASSO model, and comparing the performance of the new model to the original model with the following ratio:

$$AlgorithmReliance(var)=\frac{{MSE}_{{var}^{-}}}{{MSE}_{all\;variables}}.$$

### Latency

We calculated the per-trial latency for each neuron by finding the time from the beginning of optogenetic stimulation to the first spike of each neuron. Spikes that occurred more than 10 ms after stimulation were excluded. Neurons that were targeted with optogenetic stimulation were excluded from analysis of that trial. The latency for each neuron was the mean per-trial latency. The latency for neuron pairs was the sum of latency measure for each stimulated neuron.

To calculate latency for spontaneous ensemble activation events, we first found the median spike time for each event. We then calculated latency as the spike time of each neuron relative to the median by subtracting the median time from the spike time of each neuron.

### GCaMP Analysis

GCaMP7f and GCaMP8f rise time kinetics were taken from previous research that performed simultaneous calcium imaging and cell attached electrophysiology of neurons in mouse visual cortex [113]. These data were collected using two-photon microscopy at 112 Hz to image neurons in layer 2/3 of the visual cortex in mice. Rise kinetics were sampled from the measured single action potential rise times.

We incorporated rise time variability into our latency measurement by adding a rise time value randomly sampled from the rise time distributions found *in vivo* for each sensor in each per-trial latency measurement.

## Supporting information

S1 Fig Connection weights and distributions. (A) Representative weights and densities for connections between neuron cell types. Green boxes indicate that the connection was directly dependent on PO similarity between the neurons; neurons with more similar POs had a higher probability of being connected and had a higher probability of having a strong connection. Grey boxes indicate random connectivity. (B) Histograms of connection weights from EXC neurons to the different cell types in our simulation. (C) Histograms of connection weights from PV and VIP neurons to different cell types in our simulation. (D) Histograms of connection weights from SOM neurons to different cell types in our simulation.
(TIF)

S2 Fig Membrane voltage variability recapitulated fluctuations found *in vivo* during spontaneous activity and had robust evoked activity. (A) Representative traces of spontaneous activity of different neuron types. Excitatory resting membrane potential fluctuations were similar to those found *in vivo* in L2/3 barrel cortex neurons: *σ* = 3.45 mV (Crochet et al. [67]). Spike rates (*vertical black lines*) also matched values found *in vivo* in anesthetized mice. The gray line at the left of each trace represents −70 mV. SOM neurons had a resting potential of about −63 mV. *σ* represented the baseline membrane fluctuations over 30 ms. (B) Representative traces of excitatory neurons undergoing simulated optogenetic stimulation for 250 ms (*blue bar*). The firing rate increased during stimulation and this rate was sustained throughout the entire 250 ms. The gray line at the left of each trace represents −55 mV.
(TIF)

S3 Fig K-means clustering found orientation-selective ensembles of ~40 neurons. (A) The distribution of cluster sizes from K-means clustering when *k* = 50. Many excitatory neurons were not assigned to a neural ensemble, as indicated by the single cluster of almost 1000 neurons. Inset shows a zoomed in view of clusters with less than 120 neurons. (B) Scatter plot of cluster size versus the standard deviation of preferred orientations of neurons in that cluster. Each point represents a cluster. We focused on orientation selective ensembles in this study, so we only considered ensembles with a PO standard deviation of less than 0.25 radians. Therefore, we excluded the largest cluster from consideration when randomly selecting ensembles to train.
(TIF)

S4 Fig Ensemble characteristics were stable across ensemble sizes. (A) The correlation between neurons was consistent across ensemble size. Over all ensemble sizes, the within-cluster correlation was higher than the correlation between random neurons. The shaded region represents mean ± standard deviation (*n* = 500 neuron pairs drawn from ensembles clustered using each median cluster size). (B) The deviation in PO between neurons was consistent across ensemble size. Over all ensemble sizes, the within-cluster PO deviation was lower than the deviation between random neurons. The shaded region represents mean ± standard deviation (*n* = 500 neuron pairs drawn from ensembles clustered using each median cluster size). (C) The probability of a bidirectional connection decreased slightly as the median ensemble size increased. Over all ensemble sizes, the probability between neurons within clusters was greater than the probability between random neurons. The shaded region represents mean ± standard deviation (*n* = 500 neuron pairs drawn from ensembles clustered using each median cluster size).
(TIF)

S5 Fig Ensemble recall rate increased exponentially as pre-stimulation voltage increased. (A) Histograms of the pre-stimulation voltages for each ensemble. (B) We titrated the fold-increase in connection weights between ensemble neurons until we achieved a 5% average ensemble recall rate. (C) Example of curve fits to three different neuron pairs. The PCC was the pre-stimulation voltage when each curve intersects with a 5% recall rate. (D) *R*^2^ values for the curve fits for ensembles 1 and 2.
(TIF)

S6 Fig Baseline *V*_*m*_ and firing rates were consistent with physiological measurements during ensemble stimulation trials. (A) Average resting membrane potential for each neuron subtype in our model over 25 seconds. Our results closely match those found *in vivo* in L2/3 of the barrel cortex in mice in Neske et al. [65] and Avermann et al. [66]. Results from *in vivo* data are the mean ± s.e.m. found in those studies. (B) Average spontaneous firing rates in our model over 25 seconds resemble those found *in vivo* in anesthetized mice in Mesik et al. [63], Neill and Stryker [64], and Chen et al. [62]. Results from *in vivo* data are the mean ± s.e.m. found in those studies.
(TIF)

S7 Fig A four parameter model could accurately predict PCC of ensemble 1. (A) Best subset selection on network variables to predict PCC in ensemble 1. Black boxes indicate which variables were in the best model. * Indicates the final model chosen. (B) The *R*^2^ values for each model in (A) plateaus at 4 variables.
(TIF)

S8 Fig Graph theory parameters in ensemble 2 are similarly correlated to PCC. (A) Histogram of ensemble recall rate of 25 different pairs of neurons from ensemble 2. Each pair was stimulated 800 times. We calculated the ensemble recall rate (the fraction of trials where greater than 75% of the ensemble was activated) for each of the 25 neuron pairs. Neuron pairs with higher ensemble recall rates were considered better pattern completion neurons. (B) Probability of ensemble activation increased with the average membrane voltage of ensemble neurons before stimulation of the neuron pair. (C) The pre-stimulation voltage needed for stimulation of a neuron pair to activate an ensemble 5% of the time was significantly and negatively correlated with the overall ensemble recall rate (*p* = 1.2 × 10^−13^, *F*-test, *n* = 25 neuron pairs). Neurons with higher overall ensemble recall rates could achieve a 5% recall rate at voltages farther from threshold. (D) The correlation between each network parameter and the pattern completion capability of each neuron pair. All variables were significantly and negatively correlated with PCC, with degree and closeness centrality having the strongest correlation (from left to right: *p* = 1.8 × 10^−9^, *p* = 1.2 × 10^−8^, 2.2 × 10^−4^, 6.6 × 10^−7^, *F*-test, *n* = 25 neuron pairs).
(TIF)

S9 Fig 30 Hz stimulation does not permit recovery from previous ensemble recall. (A) We categorized the 8 stimulations for each stimulation period as either odd or even stimulations. Odd stimulations included the first stimulation of each period and had a higher recall rate compared to even stimulations, as shown in (B). (B) For both 20 Hz and 30 Hz stimulation, odd stimulation events had a higher recall rate than even events. However, the discrepancy between odd and even recall rate was greater at 30 Hz than at 20 Hz. Error bars represent standard error (*n* = 10 neuron pairs and 100 stimulations for each condition). (C) The average membrane potential of all 36 ensemble neurons during and after a successful ensemble recall event. The blue vertical line indicates the start time of a stimulation that resulted in at least 75% of ensemble neurons firing. In line with inhibitory activity shown in (E), the average ensemble membrane potential decreased abruptly at 10 ms after stimulation. This decrease was followed by a gradual rise in membrane potential back to baseline. However, at 30 Hz, the next stimulation event occurred at 33 ms, so the average ensemble voltage following a successful recall event did not have time to return to baseline. (*n* = 36 ensemble neurons during 371 recall events at 20 Hz and 132 events at 30 Hz). (D) In agreement with the average membrane potentials shown in (C), histograms of ensemble neuron pre-stimulation voltages at 20 Hz and 30 Hz show that 30 Hz even stimulation events had lower pre-stimulation voltages. This agrees with this group having the lower recall rate. Unlike at 30 Hz, even and odd stimulations at 20 Hz had similar pre-stimulation voltages, and the average was greater than 30 Hz even stimulations. Most pre-stimulation voltages were below the resting membrane potential for excitatory neurons, likely due to delayed inhibitory activity following ensemble events, as shown in (E) (*n* = 10 neuron pairs and 100 stimulations for each condition). (E) *Left*: We observed stereotypical delayed inhibitory activity following stimulation events. The average firing rate of all inhibitory neuron types increased after stimulation. The arrow denotes the inhibitory event that is shown to the right. (*n* = 10 stimulated neuron pairs over 5 stimulation periods). *Right*: The average firing rate of inhibitory neurons peaked at 10 ms following stimulation. This activity likely contributed to the low recall rate of even events at 30 Hz.
(TIF)

S1 Table Model Information. (PDF)

S2 Table *p*-values for latency to PCC correlation with 100 trials. (PDF)

## References

[1] Pérez-Ortega J, Alejandre-García T, Yuste R. Long-term stability of neuronal ensembles in mouse visual cortex. bioRxiv. 2020 Oct 28;2020.10.28.359117.

[2] Carrillo-Reid L, Yang W, Bando Y, Peterka DS, Yuste R. Imprinting and recalling cortical ensembles. Science (80-). 2016 Aug 12;353(6300):691–4. doi: 10.1126/science.aaf7560 27516599 PMC5482530

[3] MacLean JN, Watson BO, Aaron GB, Yuste R. Internal Dynamics Determine the Cortical Response to Thalamic Stimulation. Neuron. 2005 Dec 8;48(5):811–23. doi: 10.1016/j.neuron.2005.09.035 16337918

[4] Miller JEK, Ayzenshtat I, Carrillo-Reid L, Yuste R. Visual stimuli recruit intrinsically generated cortical ensembles. Proc Natl Acad Sci U S A. 2014 Sep 23;111(38):E4053–61. doi: 10.1073/pnas.1406077111 25201983 PMC4183303

[5] Cossart R, Aronov D, Yuste R. Attractor dynamics of network UP states in the neocortex. Nat 2003 4236937. 2003 May 15;423(6937):283–8. doi: 10.1038/nature01614 12748641

[6] Cai DJ, Aharoni D, Shuman T, Shobe J, Biane J, Song W, et al. A shared neural ensemble links distinct contextual memories encoded close in time. Nat 2016 5347605. 2016 May 23;534(7605):115–8. doi: 10.1038/nature17955 27251287 PMC5063500

[7] Cao VY, Ye Y, Mastwal S, Ren M, Coon M, Liu Q, et al. Motor Learning Consolidates Arc-Expressing Neuronal Ensembles in Secondary Motor Cortex. Neuron. 2015 Jun 17;86(6):1385–92. doi: 10.1016/j.neuron.2015.05.022 26051420 PMC4474764

[8] Carrillo-Reid L, Han S, Yang W, Akrouh A, Yuste R. Controlling Visually Guided Behavior by Holographic Recalling of Cortical Ensembles. Cell. 2019 Jul 11;178(2):447–457.e5. doi: 10.1016/j.cell.2019.05.045 31257030 PMC6747687

[9] Grewe BF, Gründemann J, Kitch LJ, Lecoq JA, Parker JG, Marshall JD, et al. Neural ensemble dynamics underlying a long-term associative memory. Nature. 2017 Mar 30;543(7647):670–5. doi: 10.1038/nature21682 28329757 PMC5378308

[10] Marshel JH, Kim YS, Machado TA, Quirin S, Benson B, Kadmon J, et al. Cortical layer-specific critical dynamics triggering perception. Science (80-). 2019;365(6453). doi: 10.1126/science.aaw5202 31320556 PMC6711485

[11] Hopfield JJ. Neural networks and physical systems with emergent collective computational abilities. Proc Natl Acad Sci. 1982 Apr 1;79(8):2554–8. doi: 10.1073/pnas.79.8.2554 6953413 PMC346238

[12] Guzman SJ, Schlögl A, Frotscher M, Jonas P. Synaptic mechanisms of pattern completion in the hippocampal CA3 network. Science (80-). 2016 Sep 9;353(6304):1117–23. doi: 10.1126/science.aaf1836 27609885

[13] Neunuebel JP, Knierim JJ. CA3 Retrieves Coherent Representations from Degraded Input: Direct Evidence for CA3 Pattern Completion and Dentate Gyrus Pattern Separation. Neuron. 2014 Jan 22;81(2):416–27. doi: 10.1016/j.neuron.2013.11.017 24462102 PMC3904133

[14] Abeles M. Corticonics: Neural Circuits of the Cerebral Cortex. Corticonics. 1991 Feb 22;

[15] Zatka-Haas P, Steinmetz NA, Carandini M, Harris KD. Sensory coding and the causal impact of mouse cortex in a visual decision. Elife. 2021 Jul 1;10. 10.7554/eLife.63163 PMC8324299 34328419

[16] Tang H, Schrimpf M, Lotter W, Moerman C, Paredes A, Caro JO, et al. Recurrent computations for visual pattern completion. Proc Natl Acad Sci U S A. 2018 Aug 28;115(35):8835–40. doi: 10.1073/pnas.1719397115 30104363 PMC6126774

[17] Guzowski JF, Knierim JJ, Moser EI. Ensemble Dynamics of Hippocampal Regions CA3 and CA1. Neuron. 2004 Nov 18;44(4):581–4. doi: 10.1016/j.neuron.2004.11.003 15541306

[18] Adesnik H, Abdeladim L. Probing neural codes with two-photon holographic optogenetics. Nat Neurosci 2021 2410. 2021 Aug 16;24(10):1356–66. doi: 10.1038/s41593-021-00902-9 34400843 PMC9793863

[19] Chen TW, Wardill TJ, Sun Y, Pulver SR, Renninger SL, Baohan A, et al. Ultra-sensitive fluorescent proteins for imaging neuronal activity. Nature. 2013 Jul 7;499(7458):295. doi: 10.1038/nature12354 23868258 PMC3777791

[20] Denk W, Strickler JH, Webb WW. Two-Photon Laser Scanning Fluorescence Microscopy. Science (80-). 1990;248(4951):73–6. doi: 10.1126/science.2321027 2321027

[21] Greenberg DS, Houweling AR, Kerr JND. Population imaging of ongoing neuronal activity in the visual cortex of awake rats. Nat Neurosci 2008 117. 2008 Jun 15;11(7):749–51. doi: 10.1038/nn.2140 18552841

[22] Mardinly AR, Oldenburg IA, Pégard NC, Sridharan S, Lyall EH, Chesnov K, et al. Precise multimodal optical control of neural ensemble activity. Nat Neurosci. 2018 Jun 1;21(6):881–93. doi: 10.1038/s41593-018-0139-8 29713079 PMC5970968

[23] Nikolenko V, Watson BO, Araya R, Woodruff A, Peterka DS, Yuste R. SLM microscopy: Scanless two-photon imaging and photostimulation with spatial light modulators. Front Neural Circuits. 2008 Dec 19;2(DEC):5. doi: 10.3389/neuro.04.005.2008 19129923 PMC2614319

[24] Packer AM, Russell LE, Dalgleish HWP, Häusser M. Simultaneous all-optical manipulation and recording of neural circuit activity with cellular resolution in vivo. Nat Methods. 2015 Jan 1;12(2):140–6. doi: 10.1038/nmeth.3217 25532138 PMC4933203

[25] Rickgauer JP, Deisseroth K, Tank DW. Simultaneous cellular-resolution optical perturbation and imaging of place cell firing fields. Nat Neurosci. 2014 Jan 1;17(12):1816–24. doi: 10.1038/nn.3866 25402854 PMC4459599

[26] Dalgleish HWP, Russell LE, Packer AM, Roth A, Gauld OM, Greenstreet F, et al. How many neurons are sufficient for perception of cortical activity? Elife. 2020 Oct 1;9:1–99. doi: 10.7554/eLife.58889 33103656 PMC7695456

[27] Stavisky SD, Willett FR, Wilson GH, Murphy BA, Rezaii P, Avansino DT, et al. Neural ensemble dynamics in dorsal motor cortex during speech in people with paralysis. Elife. 2019 Dec 1;8. doi: 10.7554/eLife.46015 31820736 PMC6954053

[28] Carrillo-Reid L, Miller J-EK, Hamm JP, Jackson J, Yuste R. Endogenous Sequential Cortical Activity Evoked by Visual Stimuli. 2015; doi: 10.1523/JNEUROSCI.5214-14.2015 26063915 PMC4461687

[29] Harvey CD, Coen P, Tank DW. Choice-specific sequences in parietal cortex during a virtual-navigation decision task. Nat 2012 4847392. 2012 Mar 14;484(7392):62–8. doi: 10.1038/nature10918 22419153 PMC3321074

[30] Noguchi A, Huszár R, Morikawa S, Buzsáki G, Ikegaya Y. Inhibition allocates spikes during hippocampal ripples. Nat Commun 2022 131. 2022 Mar 11;13(1):1–14. doi: 10.1038/s41467-022-28890-9 35277500 PMC8917132

[31] Louie K, Wilson MA. Temporally structured replay of awake hippocampal ensemble activity during rapid eye movement sleep. Neuron. 2001;29(1):145–56. doi: 10.1016/s0896-6273(01)00186-6 11182087

[32] Boyden ES, Zhang F, Bamberg E, Nagel G, Deisseroth K. Millisecond-timescale, genetically targeted optical control of neural activity. Nat Neurosci 2005 89. 2005 Aug 14;8(9):1263–8. doi: 10.1038/nn1525 16116447

[33] Yizhar O, Fenno LE, Davidson TJ, Mogri M, Deisseroth K. Optogenetics in Neural Systems. Neuron. 2011 Jul 14;71(1):9–34. doi: 10.1016/j.neuron.2011.06.004 21745635

[34] Bassett DS, Bullmore ET. Small-World Brain Networks Revisited. Neuroscientist. 2017 Oct 21;23(5):499–516. doi: 10.1177/1073858416667720 27655008 PMC5603984

[35] Del Ferraro G, Moreno A, Min B, Morone F, Pérez-Ramírez Ú, Pérez-Cervera L, et al. Finding influential nodes for integration in brain networks using optimal percolation theory. Nat Commun. 2018 Dec 1;9(1):1–12. 29891915 10.1038/s41467-018-04718-3 PMC5995874

[36] Du Y, Gao C, Hu Y, Mahadevan S, Deng Y. A new method of identifying influential nodes in complex networks based on TOPSIS. Phys A Stat Mech its Appl. 2014 Apr 1;399:57–69.

[37] Lynn CW, Bassett DS. The physics of brain network structure, function and control. Vol. 1, Nature Reviews Physics. Springer Nature; 2019. p. 318–32.

[38] Sporns O, Honey CJ, Kötter R. Identification and classification of hubs in brain networks. PLoS One. 2007 Oct 17;2(10):1049. doi: 10.1371/journal.pone.0001049 17940613 PMC2013941

[39] Stam CJ, Reijneveld JC. Graph theoretical analysis of complex networks in the brain. Vol. 1, Nonlinear Biomedical Physics. BioMed Central; 2007. p. 1–19. 10.1186/1753-4631-1-3 PMC1976403 17908336

[40] Zhao J, Wang Y, Deng Y. Identifying influential nodes in complex networks from global perspective. Chaos, Solitons and Fractals. 2020 Apr 1;133:109637.

[41] Alstott J, Breakspear M, Hagmann P, Cammoun L, Sporns O. Modeling the Impact of Lesions in the Human Brain. PLOS Comput Biol. 2009 Jun;5(6):e1000408. doi: 10.1371/journal.pcbi.1000408 19521503 PMC2688028

[42] Betzel RF, Gu S, Medaglia JD, Pasqualetti F, Bassett DS. Optimally controlling the human connectome: The role of network topology. Sci Rep. 2016 Jul 29;6(1):1–14. 27468904 10.1038/srep30770 PMC4965758

[43] Betzel RF, Bassett DS. Multi-scale brain networks. Neuroimage. 2017 Oct 15;160:73–83. doi: 10.1016/j.neuroimage.2016.11.006 27845257 PMC5695236

[44] Gu S, Pasqualetti F, Cieslak M, Telesford QK, Yu AB, Kahn AE, et al. Controllability of structural brain networks. Nat Commun. 2015 Oct 1;6(1):1–10. doi: 10.1038/ncomms9414 26423222 PMC4600713

[45] Kim JZ, Soffer JM, Kahn AE, Vettel JM, Pasqualetti F, Bassett DS. Role of graph architecture in controlling dynamical networks with applications to neural systems. Nat Phys. 2018;14(1):91–8. doi: 10.1038/nphys4268 29422941 PMC5798649

[46] Betzel RF, Woodid KC, Angeloni C, Geffen MN, Bassettid DS. Stability of spontaneous, correlated activity in mouse auditory cortex. 2019; doi: 10.1371/journal.pcbi.1007360 31815941 PMC6968873

[47] Paninski L, Cunningham JP. Neural data science: accelerating the experiment-analysis-theory cycle in large-scale neuroscience. Curr Opin Neurobiol. 2018 Jun 1;50:232–41. doi: 10.1016/j.conb.2018.04.007 29738986

[48] Berens P, Freeman J, Deneux T, Chenkov N, McColgan T, Speiser A, et al. Community-based benchmarking improves spike rate inference from two-photon calcium imaging data. PLOS Comput Biol. 2018 May 1;14(5):e1006157. doi: 10.1371/journal.pcbi.1006157 29782491 PMC5997358

[49] Buzsáki G, Anastassiou CA, Koch C. The origin of extracellular fields and currents—EEG, ECoG, LFP and spikes. Nat Rev Neurosci 2012 136. 2012 May 18;13(6):407–20. doi: 10.1038/nrn3241 22595786 PMC4907333

[50] Obien MEJ, Deligkaris K, Bullmann T, Bakkum DJ, Frey U. Revealing neuronal function through microelectrode array recordings. Front Neurosci. 2015;9(JAN):423. doi: 10.3389/fnins.2014.00423 25610364 PMC4285113

[51] Keller AJ, Dipoppa M, Roth MM, Caudill MS, Ingrosso A, Miller KD, et al. A Disinhibitory Circuit for Contextual Modulation in Primary Visual Cortex. Neuron. 2020 Dec 23;108(6):1181–1193.e8. doi: 10.1016/j.neuron.2020.11.013 33301712 PMC7850578

[52] Wilmes KA, Clopath C. Inhibitory microcircuits for top-down plasticity of sensory representations. Nat Commun 2019 101. 2019 Nov 7;10(1):1–10. doi: 10.1038/s41467-019-12972-2 31699994 PMC6838080

[53] Litwin-Kumar A, Rosenbaum R, Doiron B. Inhibitory stabilization and visual coding in cortical circuits with multiple interneuron subtypes. J Neurophysiol. 2016 Mar 1;115(3):1399–409. doi: 10.1152/jn.00732.2015 26740531 PMC4808082

[54] Sadeh S, Clopath C. Patterned perturbation of inhibition can reveal the dynamical structure of neural processing. Elife. 2020 Feb 1;9. doi: 10.7554/eLife.52757 32073400 PMC7180056

[55] Sadeh S, Clopath C. Theory of neuronal perturbome in cortical networks. Proc Natl Acad Sci U S A. 2020 Oct 27;117(43):26966–76. doi: 10.1073/pnas.2004568117 33055215 PMC7604497

[56] Pfeffer CK, Xue M, He M, Huang ZJ, Scanziani M. Inhibition of inhibition in visual cortex: The logic of connections between molecularly distinct interneurons. Nat Neurosci. 2013 Aug;16(8):1068–76. doi: 10.1038/nn.3446 23817549 PMC3729586

[57] Cossell L, Iacaruso MF, Muir DR, Houlton R, Sader EN, Ko H, et al. Functional organization of excitatory synaptic strength in primary visual cortex. Nature. 2015 Feb 19;518(7539):399–403. doi: 10.1038/nature14182 25652823 PMC4843963

[58] Ko H, Hofer SB, Pichler B, Buchanan KA, Sjöström PJ, Mrsic-Flogel TD. Functional specificity of local synaptic connections in neocortical networks. Nature. 2011 May 5;473(7345):87–91. doi: 10.1038/nature09880 21478872 PMC3089591

[59] Lee WCA, Bonin V, Reed M, Graham BJ, Hood G, Glattfelder K, et al. Anatomy and function of an excitatory network in the visual cortex. Nat 2016 5327599. 2016 Mar 28;532(7599):370–4. doi: 10.1038/nature17192 27018655 PMC4844839

[60] Znamenskiy P, Kim M-H, Muir D, Iacaruso MF, Hofer S, Mrsic-Flogel T. Functional selectivity and specific connectivity of inhibitory neurons in primary visual cortex. bioRxiv. 2018 Apr 4;294835.

[61] Buzsáki G, Mizuseki K. The log-dynamic brain: how skewed distributions affect network operations. Nat Rev Neurosci 2014 154. 2014 Feb 26;15(4):264–78. doi: 10.1038/nrn3687 24569488 PMC4051294

[62] Chen N, Sugihara H, Sur M. An acetylcholine-activated microcircuit drives temporal dynamics of cortical activity. Nat Neurosci. 2015 Jun 28;18(6):892–902. doi: 10.1038/nn.4002 25915477 PMC4446146

[63] Mesik L, Ma WP, Li LY, Ibrahim LA, Huang ZJ, Zhang L, et al. Functional response properties of VIP-expressing inhibitory neurons in mouse visual and auditory cortex. Front Neural Circuits. 2015 May 22;9(May):22. doi: 10.3389/fncir.2015.00022 26106301 PMC4460767

[64] Niell CM, Stryker MP. Highly selective receptive fields in mouse visual cortex. J Neurosci. 2008 Jul 23;28(30):7520–36. doi: 10.1523/JNEUROSCI.0623-08.2008 18650330 PMC3040721

[65] Neske GT, Patrick SL, Connors BW. Contributions of Diverse Excitatory and Inhibitory Neurons to Recurrent Network Activity in Cerebral Cortex. J Neurosci. 2015 Jan 21;35(3):1089–105. doi: 10.1523/JNEUROSCI.2279-14.2015 25609625 PMC4300319

[66] Avermann M, Tomm C, Mateo C, Gerstner W, Petersen CCH. Microcircuits of excitatory and inhibitory neurons in layer 2/3 of mouse barrel cortex. J Neurophysiol. 2012 Jun 1;107(11):3116–34. doi: 10.1152/jn.00917.2011 22402650

[67] Crochet S, Poulet JFA, Kremer Y, Petersen CCH. Synaptic mechanisms underlying sparse coding of active touch. Neuron. 2011;69(6):1160–75. doi: 10.1016/j.neuron.2011.02.022 21435560

[68] Wang Y, Liu YZ, Wang SY, Wang Z. In vivo whole-cell recording with high success rate in anaesthetized and awake mammalian brains. Mol Brain. 2016 Sep 29;9(1):1–14. doi: 10.1186/s13041-016-0266-7 27680101 PMC5041312

[69] Chettih SN, Harvey CD. Single-neuron perturbations reveal feature-specific competition in V1. Nature. 2019 Mar 21;567(7748):334–40. doi: 10.1038/s41586-019-0997-6 30842660 PMC6682407

[70] Kwan AC, Dan Y. Dissection of cortical microcircuits by single-neuron stimulation in vivo. Curr Biol. 2012 Aug 21;22(16):1459–67. doi: 10.1016/j.cub.2012.06.007 22748320 PMC3467311

[71] Billeh YN, Cai B, Gratiy SL, Dai K, Iyer R, Gouwens NW, et al. Systematic Integration of Structural and Functional Data into Multi-scale Models of Mouse Primary Visual Cortex. Neuron. 2020 May 6;106(3):388–403.e18. doi: 10.1016/j.neuron.2020.01.040 32142648

[72] Peron S, Pancholi R, Voelcker B, Wittenbach JD, Ólafsdóttir HF, Freeman J, et al. Recurrent interactions in local cortical circuits. Nature. 2020 Mar 4;579(7798):256–9. doi: 10.1038/s41586-020-2062-x 32132709 PMC8092186

[73] Douglas RJ, Koch C, Mahowald M, Martin KAC, Suarez HH. Recurrent Excitation in Neocortical Circuits. Science (80-). 1995;269(5226):981–5. doi: 10.1126/science.7638624 7638624

[74] Douglas RJ, Martin KAC. Recurrent neuronal circuits in the neocortex. Curr Biol. 2007 Jul 3;17(13):R496–500. doi: 10.1016/j.cub.2007.04.024 17610826

[75] Alejandre-García T, Kim S, Pérez-Ortega J, Yuste R. Intrinsic excitability mechanisms of neuronal ensemble formation. Elife. 2022;11. doi: 10.7554/eLife.77470 35506662 PMC9197391

[76] Bando Y, Grimm C, Cornejo VH, Yuste R. Genetic voltage indicators. BMC Biol. 2019 Sep 12;17(1):1–12. 31514747 10.1186/s12915-019-0682-0 PMC6739974

[77] Wu CT, Haggerty D, Kemere C, Ji D. Hippocampal awake replay in fear memory retrieval. Nat Neurosci 2017 204. 2017 Feb 20;20(4):571–80. doi: 10.1038/nn.4507 28218916 PMC5373994

[78] Akhlaghpour H, Wiskerke J, Choi JY, Taliaferro JP, Au J, Witten IB. Dissociated sequential activity and stimulus encoding in the dorsomedial striatum during spatial working memory. Elife. 2016 Sep 16;5(September2016). doi: 10.7554/eLife.19507 27636864 PMC5053805

[79] Fujisawa S, Amarasingham A, Harrison MT, Buzsáki G. Behavior-dependent short-term assembly dynamics in the medial prefrontal cortex. Nat Neurosci 2008 117. 2008 May 30;11(7):823–33. doi: 10.1038/nn.2134 18516033 PMC2562676

[80] Pastalkova E, Itskov V, Amarasingham A, Buzsáki G. Internally generated cell assembly sequences in the rat hippocampus. Science (80-). 2008 Sep 5;321(5894):1322–7. doi: 10.1126/science.1159775 18772431 PMC2570043

[81] Zhang Y, Rózsa M, Liang Y, Bushey D, Wei Z, Zheng J, et al. Fast and sensitive GCaMP calcium indicators for imaging neural populations. Nature. 2023 Mar 15;1–8. doi: 10.1038/s41586-023-05828-9 36922596 PMC10060165

[82] Carrillo-Reid L, Han S, O DA, Taralova E, Yuste R. Identification of Pattern Completion Neurons in Neuronal Ensembles using Probabilistic Graphical Models. J Neurosci. 2021;(August). doi: 10.1523/JNEUROSCI.0051-21.2021 34413204 PMC8513696

[83] Liu Y, Wei B, Du Y, Xiao F, Deng Y. Identifying influential spreaders by weight degree centrality in complex networks. Chaos, Solitons and Fractals. 2016 May 1;86:1–7.

[84] Malliaros FD, Rossi MEG, Vazirgiannis M. Locating influential nodes in complex networks. Sci Rep. 2016 Jan 18;6(1):1–10. 26776455 10.1038/srep19307 PMC4725982

[85] Sheikhahmadi A, Nematbakhsh MA, Shokrollahi A. Improving detection of influential nodes in complex networks. Phys A Stat Mech its Appl. 2015 Jul 21;436:833–45.

[86] Wang M, Li W, Guo Y, Peng X, Li Y. Identifying influential spreaders in complex networks based on improved k-shell method. Phys A Stat Mech its Appl. 2020 Sep 15;554:124229.

[87] Pavlopoulos GA, Secrier M, Moschopoulos CN, Soldatos TG, Kossida S, Aerts J, et al. Using graph theory to analyze biological networks. Vol. 4, BioData Mining. BioMed Central; 2011. p. 10. doi: 10.1186/1756-0381-4-10 21527005 PMC3101653

[88] Guo WF, Zhang SW, Shi QQ, Zhang CM, Zeng T, Chen L. A novel algorithm for finding optimal driver nodes to target control complex networks and its applications for drug targets identification. BMC Genomics. 2018 Jan 19;19(S1):924. doi: 10.1186/s12864-017-4332-z 29363426 PMC5780855

[89] Kempe D, Kleinberg J, Tardos E. Maximizing the Spread of Influence through a Social Network. 2003.

[90] Robinaugh DJ, Millner AJ, McNally RJ. Identifying highly influential nodes in the complicated grief network. J Abnorm Psychol. 2016 Aug 1;125(6):747–57. doi: 10.1037/abn0000181 27505622 PMC5060093

[91] Liu YY, Slotine JJ, Barabási AL. Controllability of complex networks. Nature. 2011 May 12;473(7346):167–73. doi: 10.1038/nature10011 21562557

[92] Pasqualetti F, Zampieri S, Bullo F. Controllability metrics, limitations and algorithms for complex networks. Proc Am Control Conf. 2014;3287–92.

[93] Vlachos I, Aertsen A, Kumar A. Beyond Statistical Significance: Implications of Network Structure on Neuronal Activity. PLOS Comput Biol. 2012 Jan;8(1):e1002311. doi: 10.1371/journal.pcbi.1002311 22291581 PMC3266872

[94] Yuan Z, Zhao C, Di Z, Wang WX, Lai YC. Exact controllability of complex networks. Nat Commun 2013 41. 2013 Sep 12;4(1):1–9. doi: 10.1038/ncomms3447 24025746 PMC3945876

[95] Bassett DS, Zurn P, Gold JI. Network Models in Neuroscience. In: The Neocortex. 2020.

[96] Bullmore E, Sporns O. Complex brain networks: graph theoretical analysis of structural and functional systems. Nat Rev Neurosci 2009 103. 2009 Feb 4;10(3):186–98. doi: 10.1038/nrn2575 19190637

[97] Zuo XN, Ehmke R, Mennes M, Imperati D, Castellanos FX, Sporns O, et al. Network centrality in the human functional connectome. Cereb Cortex. 2012 Aug;22(8):1862–75. doi: 10.1093/cercor/bhr269 21968567

[98] Morone F, Roth K, Min B, Stanley HE, Makse HA. Model of brain activation predicts the neural collective influence map of the brain. Proc Natl Acad Sci U S A. 2017 Apr 11;114(15):3849–54. doi: 10.1073/pnas.1620808114 28351973 PMC5393219

[99] Roebroeck A, Formisano E, Goebel R. Mapping directed influence over the brain using Granger causality and fMRI. Neuroimage. 2005 Mar 1;25(1):230–42. doi: 10.1016/j.neuroimage.2004.11.017 15734358

[100] Mann K, Gallen CL, Clandinin TR. Whole-Brain Calcium Imaging Reveals an Intrinsic Functional Network in Drosophila. Curr Biol. 2017 Aug 7;27(15):2389–2396.e4. doi: 10.1016/j.cub.2017.06.076 28756955 PMC5967399

[101] Kobayashi R, Kurita S, Kurth A, Kitano K, Mizuseki K, Diesmann M, et al. Reconstructing neuronal circuitry from parallel spike trains. Nat Commun 2019 101. 2019 Oct 2;10(1):1–13. doi: 10.1038/s41467-019-12225-2 31578320 PMC6775109

[102] Sadovsky AJ, MacLean JN. Scaling of Topologically Similar Functional Modules Defines Mouse Primary Auditory and Somatosensory Microcircuitry. J Neurosci. 2013 Aug 28;33(35):14048–60. doi: 10.1523/JNEUROSCI.1977-13.2013 23986241 PMC3756753

[103] Cramer J V., Gesierich B, Roth S, Dichgans M, Düring M, Liesz A. In vivo widefield calcium imaging of the mouse cortex for analysis of network connectivity in health and brain disease. Neuroimage. 2019 Oct 1;199:570–84. doi: 10.1016/j.neuroimage.2019.06.014 31181333

[104] Stetter O, Battaglia D, Soriano J, Geisel T. Model-Free Reconstruction of Excitatory Neuronal Connectivity from Calcium Imaging Signals. PLOS Comput Biol. 2012 Aug;8(8):e1002653. doi: 10.1371/journal.pcbi.1002653 22927808 PMC3426566

[105] Garofalo M, Nieus T, Massobrio P, Martinoia S. Evaluation of the Performance of Information Theory-Based Methods and Cross-Correlation to Estimate the Functional Connectivity in Cortical Networks. PLoS One. 2009 Aug 4;4(8):e6482. doi: 10.1371/journal.pone.0006482 19652720 PMC2715865

[106] Nigam S, Shimono M, Ito S, Yeh FC, Timme N, Myroshnychenko M, et al. Rich-Club Organization in Effective Connectivity among Cortical Neurons. J Neurosci. 2016 Jan 20;36(3):670–84. doi: 10.1523/JNEUROSCI.2177-15.2016 26791200 PMC4719009

[107] Patel TP, Man K, Firestein BL, Meaney DF. Automated quantification of neuronal networks and single-cell calcium dynamics using calcium imaging. J Neurosci Methods. 2015 Mar 1;243:26–38. doi: 10.1016/j.jneumeth.2015.01.020 25629800 PMC5553047

[108] Kelly RC, Smith MA, Samonds JM, Kohn A, Bonds AB, Movshon JA, et al. Comparison of Recordings from Microelectrode Arrays and Single Electrodes in the Visual Cortex. J Neurosci. 2007 Jan 10;27(2):261–4. doi: 10.1523/JNEUROSCI.4906-06.2007 17215384 PMC3039847

[109] Barz F, Livi A, Lanzilotto M, Maranesi M, Bonini L, Paul O, et al. Versatile, modular 3D microelectrode arrays for neuronal ensemble recordings: from design to fabrication, assembly, and functional validation in non-human primates. J Neural Eng. 2017 Mar 29;14(3). doi: 10.1088/1741-2552/aa5a90 28102825

[110] Franke F, Jäckel D, Dragas J, Müller J, Radivojevic M, Bakkum D, et al. High-density microelectrode array recordings and real-time spike sorting for closed-loop experiments: An emerging technology to study neural plasticity. Front Neural Circuits. 2012 Dec 2;0(DEC):105. doi: 10.3389/fncir.2012.00105 23267316 PMC3526803

[111] Bakkum DJ, Chao ZC, Potter SM. Long-Term Activity-Dependent Plasticity of Action Potential Propagation Delay and Amplitude in Cortical Networks. PLoS One. 2008 May 7;3(5):e2088. doi: 10.1371/journal.pone.0002088 18461127 PMC2324202

[112] Huang L, Ledochowitsch P, Knoblich U, Lecoq J, Murphy GJ, Reid RC, et al. Relationship between simultaneously recorded spiking activity and fluorescence signal in gcamp6 transgenic mice. Elife. 2021 Mar 1;10. doi: 10.7554/eLife.51675 33683198 PMC8060029

[113] Zhang Y, Rózsa M, Bushey D, Zheng J, Reep D, Liang Y, et al. jGCaMP8 Fast Genetically Encoded Calcium Indicators [Internet]. figshare; 2020. Available from: https://janelia.figshare.com/articles/online_resource/jGCaMP8_Fast_Genetically_Encoded_Calcium_Indicators/13148243

[114] Adam Y, Kim JJ, Lou S, Zhao Y, Xie ME, Brinks D, et al. Voltage imaging and optogenetics reveal behaviour-dependent changes in hippocampal dynamics. Nature. 2019 May 1;569(7756):413–7. doi: 10.1038/s41586-019-1166-7 31043747 PMC6613938

[115] Beck C, Zhang D, Gong Y. Enhanced genetically encoded voltage indicators advance their applications in neuroscience. Curr Opin Biomed Eng. 2019 Dec 1;12:111–7. doi: 10.1016/j.cobme.2019.10.010 32864526 PMC7449513

[116] Gong Y, Huang C, Li JZ, Grewe BF, Zhang Y, Eismann S, et al. High-speed recording of neural spikes in awake mice and flies with a fluorescent voltage sensor. Science (80-). 2015 Dec 11;350(6266):1361. doi: 10.1126/science.aab0810 26586188 PMC4904846

[117] Kim TH, Schnitzer MJ. Fluorescence imaging of large-scale neural ensemble dynamics. Cell. 2022 Jan 6;185(1):9–41. doi: 10.1016/j.cell.2021.12.007 34995519 PMC8849612

[118] Bi X, Beck C, Gong Y. Genetically Encoded Fluorescent Indicators for Imaging Brain Chemistry. Biosens 2021, Vol 11, Page 116. 2021 Apr 11;11(4):116. doi: 10.3390/bios11040116 33920418 PMC8069469

[119] Neumann AR, Raedt R, Steenland HW, Sprengers M, Bzymek K, Navratilova Z, et al. Involvement of fast-spiking cells in ictal sequences during spontaneous seizures in rats with chronic temporal lobe epilepsy. Brain. 2017 Sep 1;140(9):2355–69. doi: 10.1093/brain/awx179 29050390 PMC6248724

[120] Liu J, Baraban SC. Network Properties Revealed during Multi-Scale Calcium Imaging of Seizure Activity in Zebrafish. eNeuro. 2019 Jan 1;6(1). doi: 10.1523/ENEURO.0041-19.2019 30895220 PMC6424556

[121] Wenzel M, Hamm JP, Peterka DS, Yuste R. Reliable and elastic propagation of cortical seizures in vivo. Cell Rep. 2017 Jun 6;19(13):2681. 28658617 10.1016/j.celrep.2017.05.090 PMC5551439

[122] Berditchevskaia A, Cazé RD, Schultz SR. Performance in a GO/NOGO perceptual task reflects a balance between impulsive and instrumental components of behaviour. Sci Rep. 2016 Jun 7;6. 27272438 10.1038/srep27389 PMC4895381

[123] Liu D, Deng J, Zhang Z, Zhang ZY, Sun YG, Yang T, et al. Orbitofrontal control of visual cortex gain promotes visual associative learning. Nat Commun 2020 111. 2020 Jun 3;11(1):1–14. doi: 10.1038/s41467-020-16609-7 32493971 PMC7270099

[124] Grewe BF, Langer D, Kasper H, Kampa BM, Helmchen F. High-speed in vivo calcium imaging reveals neuronal network activity with near-millisecond precision. Nat Methods 2010 75. 2010 Apr 18;7(5):399–405. doi: 10.1038/nmeth.1453 20400966

[125] Katona G, Szalay G, Maák P, Kaszás A, Veress M, Hillier D, et al. Fast two-photon in vivo imaging with three-dimensional random-access scanning in large tissue volumes. Nat Methods 2012 92. 2012 Jan 8;9(2):201–8. doi: 10.1038/nmeth.1851 22231641

[126] Botcherby EJ, Smith CW, Kohl MM, Deb́arre D, Booth MJ, Juškaitis R, et al. Aberration-free three-dimensional multiphoton imaging of neuronal activity at kHz rates. Proc Natl Acad Sci U S A. 2012 Feb 21;109(8):2919–24. doi: 10.1073/pnas.1111662109 22315405 PMC3286923

[127] Carrillo-Reid L, Yuste R. Playing the piano with the cortex: role of neuronal ensembles and pattern completion in perception and behavior. Curr Opin Neurobiol. 2020 Oct 1;64:89–95. doi: 10.1016/j.conb.2020.03.014 32320944 PMC8006069

[128] Hebb D. The organization of behavior; a neuropsychological theory. Wiley; 1949.

[129] Clopath C, Büsing L, Vasilaki E, Gerstner W. Connectivity reflects coding: a model of voltage-based STDP with homeostasis. Nat Neurosci 2010 133. 2010 Jan 24;13(3):344–52. 10.1038/nn.2479 20098420

[130] Vogels TP, Sprekeler H, Zenke F, Clopath C, Gerstner W. Inhibitory plasticity balances excitation and inhibition in sensory pathways and memory networks. Science (80-). 2011;334(6062):1569–73. doi: 10.1126/science.1211095 22075724

[131] Lin JY. A User’s Guide to Channelrhodopsin Variants: Features, Limitations and Future Developments. Exp Physiol. 2011 Jan 1;96(1):19. doi: 10.1113/expphysiol.2009.051961 20621963 PMC2995811

[132] Feldmeyer D, Lübke J, Sakmann B. Efficacy and connectivity of intracolumnar pairs of layer 2/3 pyramidal cells in the barrel cortex of juvenile rats. J Physiol. 2006 Sep;575(Pt 2):583–602. doi: 10.1113/jphysiol.2006.105106 16793907 PMC1819447

[133] Wehr M, Zador AM. Balanced inhibition underlies tuning and sharpens spike timing in auditory cortex. Nat 2003 4266965. 2003 Nov 27;426(6965):442–6. doi: 10.1038/nature02116 14647382

