# Supplementary figures and images for: Identifying properties of pattern completion neurons in a computational model of the visual cortex
Source: PLoS Comput Biol. 2023 Jun 6;19(6):e1011167. doi: 10.1371/journal.pcbi.1011167 (PMC10275485; doi:10.1371/journal.pcbi.1011167)

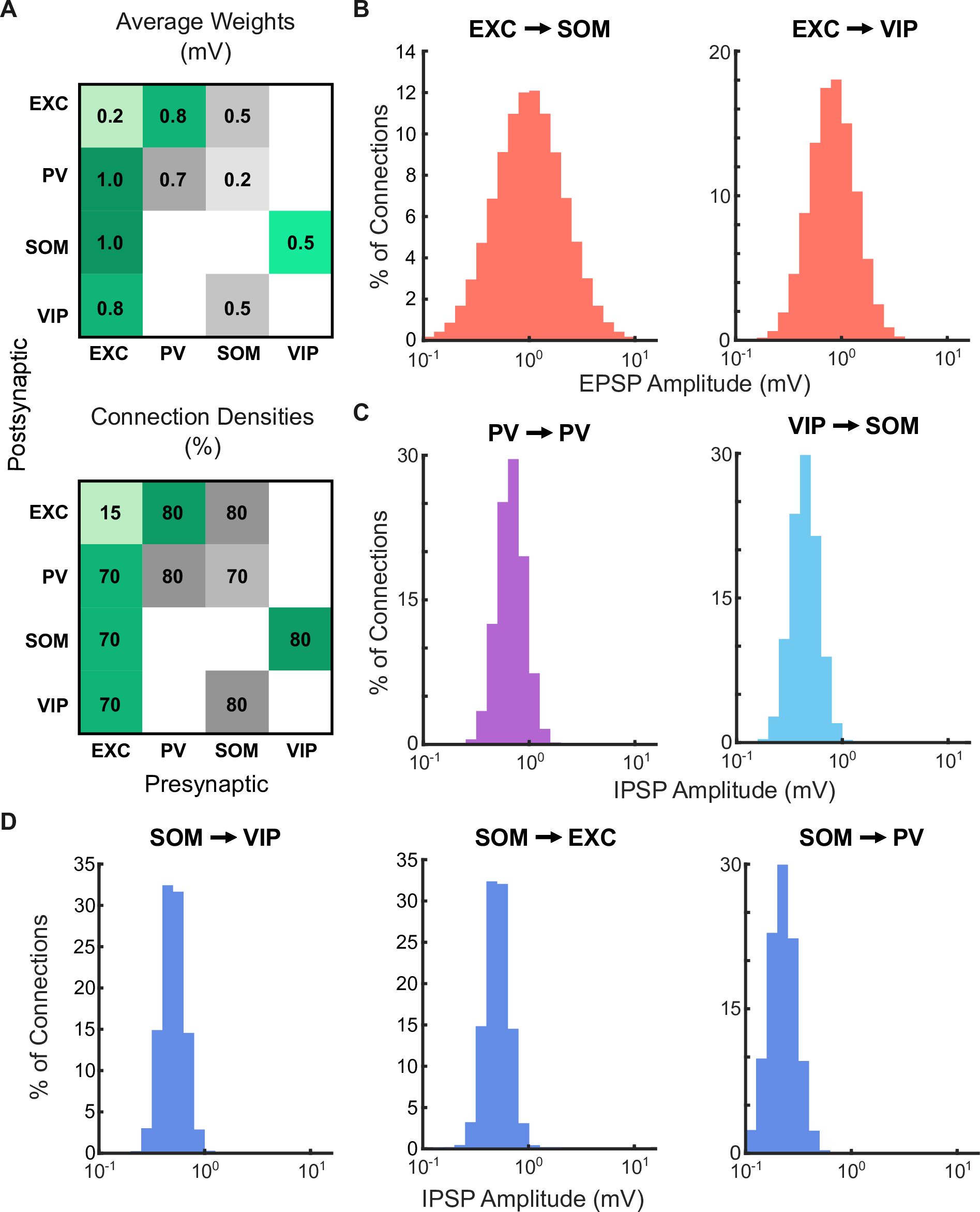

Supplement: S1 Fig — (A) Representative weights and densities for connections between neuron cell types. Green boxes indicate that the connection was directly dependent on PO similarity between the neurons; neurons with more similar POs had a higher probability of being connected and had a higher probability of having a strong connection. Grey boxes indicate random connectivity. (B) Histograms of connection weights from EXC neurons to the different cell types in our simulation. (C) Histograms of connection weights from PV and VIP neurons to different cell types in our simulation. (D) Histograms of connection weights from SOM neurons to different cell types in our simulation. (TIF) [file pcbi.1011167.s001.tif]

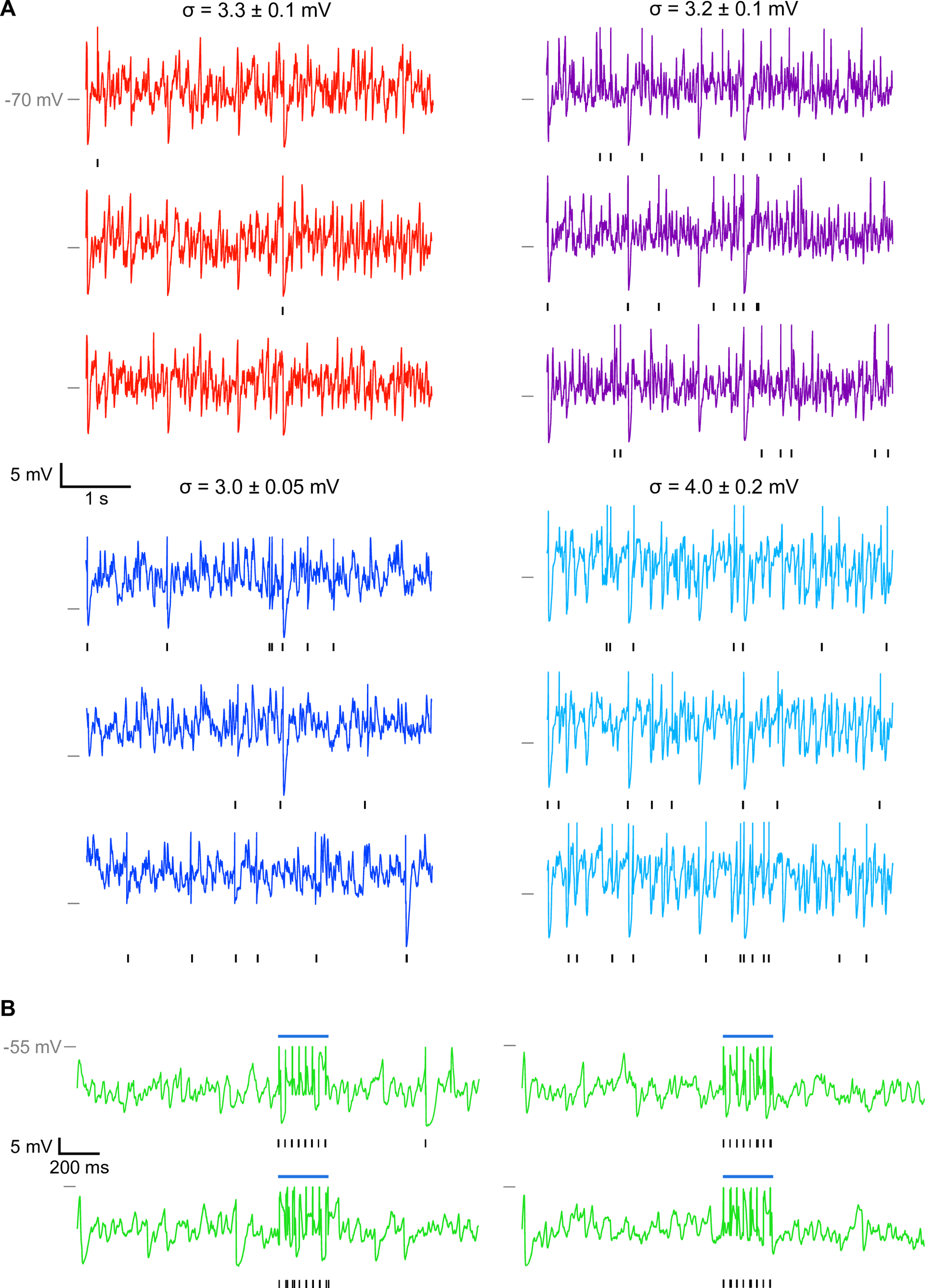

Supplement: S2 Fig — (A) Representative traces of spontaneous activity of different neuron types. Excitatory resting membrane potential fluctuations were similar to those found in vivo in L2/3 barrel cortex neurons: σ = 3.45 mV (Crochet et al. [67]). Spike rates (vertical black lines) also matched values found in vivo in anesthetized mice. The gray line at the left of each trace represents −70 mV. SOM neurons had a resting potential of about −63 mV. σ represented the baseline membrane fluctuations over 30 ms. (B) Representative traces of excitatory neurons undergoing simulated optogenetic stimulation for 250 ms (blue bar). The firing rate increased during stimulation and this rate was sustained throughout the entire 250 ms. The gray line at the left of each trace represents −55 mV. (TIF) [file pcbi.1011167.s002.tif]

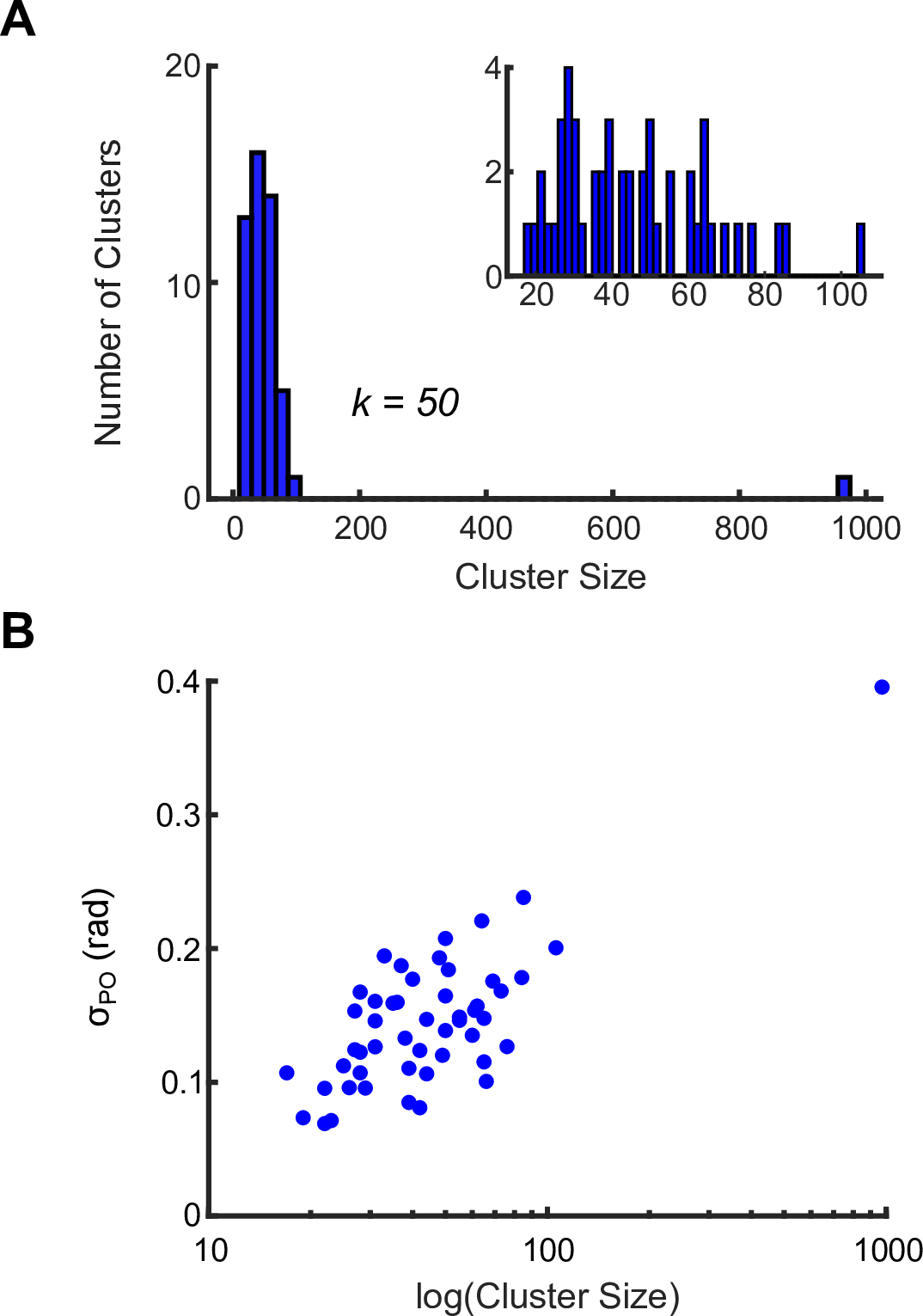

Supplement: S3 Fig — (A) The distribution of cluster sizes from K-means clustering when k = 50. Many excitatory neurons were not assigned to a neural ensemble, as indicated by the single cluster of almost 1000 neurons. Inset shows a zoomed in view of clusters with less than 120 neurons. (B) Scatter plot of cluster size versus the standard deviation of preferred orientations of neurons in that cluster. Each point represents a cluster. We focused on orientation selective ensembles in this study, so we only considered ensembles with a PO standard deviation of less than 0.25 radians. Therefore, we excluded the largest cluster from consideration when randomly selecting ensembles to train. (TIF) [file pcbi.1011167.s003.tif]

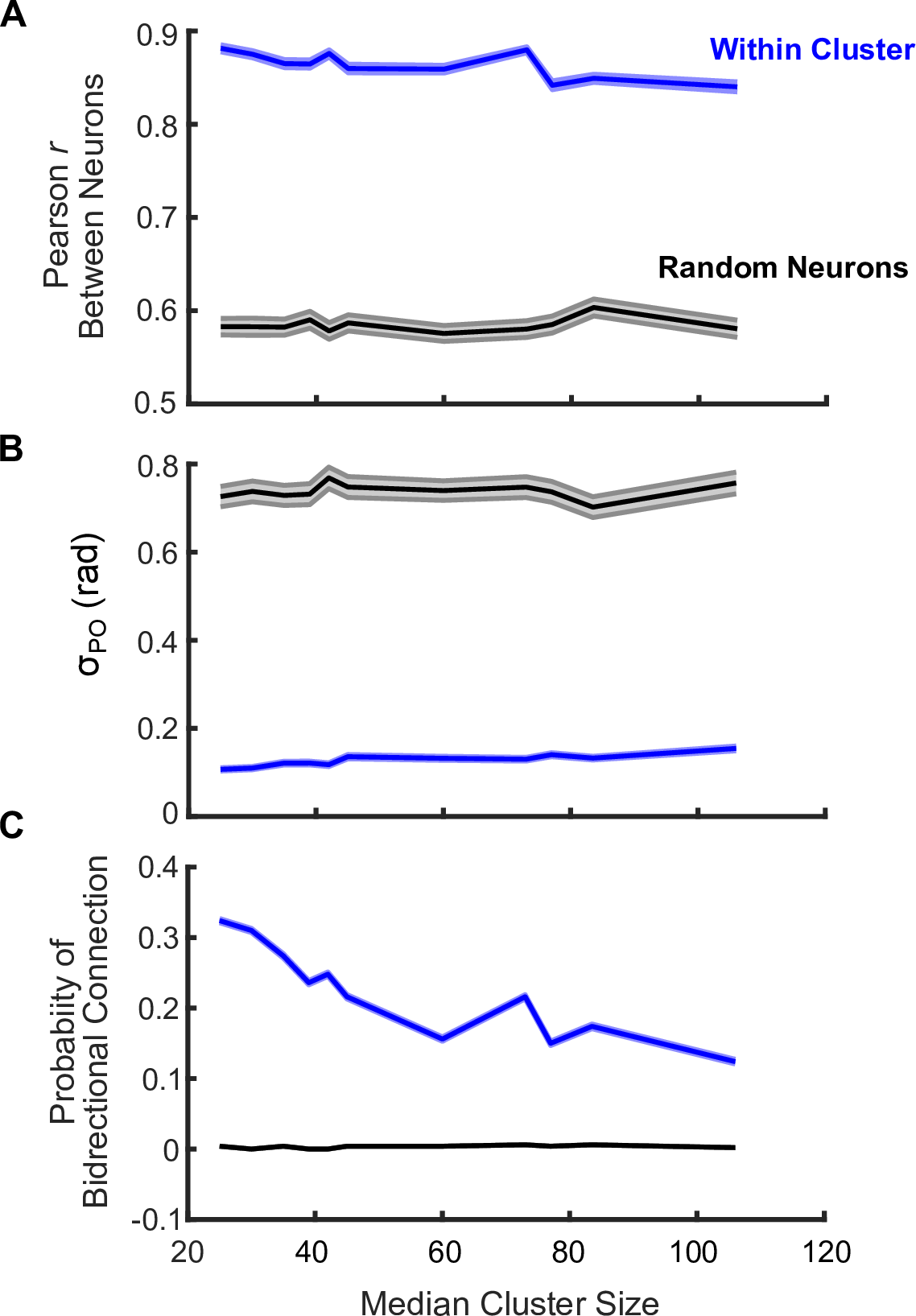

Supplement: S4 Fig — (A) The correlation between neurons was consistent across ensemble size. Over all ensemble sizes, the within-cluster correlation was higher than the correlation between random neurons. The shaded region represents mean ± standard deviation (n = 500 neuron pairs drawn from ensembles clustered using each median cluster size). (B) The deviation in PO between neurons was consistent across ensemble size. Over all ensemble sizes, the within-cluster PO deviation was lower than the deviation between random neurons. The shaded region represents mean ± standard deviation (n = 500 neuron pairs drawn from ensembles clustered using each median cluster size). (C) The probability of a bidirectional connection decreased slightly as the median ensemble size increased. Over all ensemble sizes, the probability between neurons within clusters was greater than the probability between random neurons. The shaded region represents mean ± standard deviation (n = 500 neuron pairs drawn from ensembles clustered using each median cluster size). (TIF) [file pcbi.1011167.s004.tif]

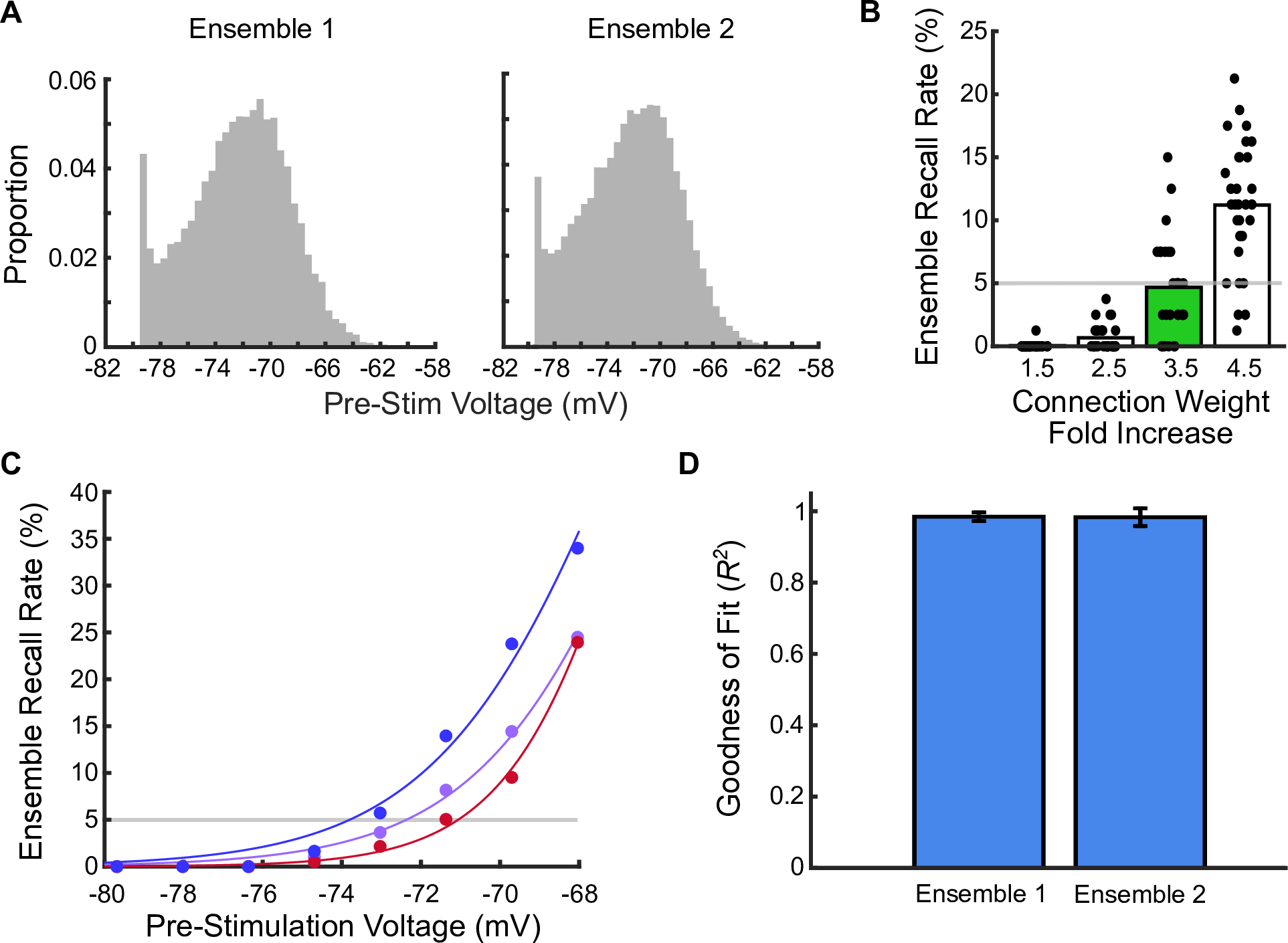

Supplement: S5 Fig — (A) Histograms of the pre-stimulation voltages for each ensemble. (B) We titrated the fold-increase in connection weights between ensemble neurons until we achieved a 5% average ensemble recall rate. (C) Example of curve fits to three different neuron pairs. The PCC was the pre-stimulation voltage when each curve intersects with a 5% recall rate. (D) R2 values for the curve fits for ensembles 1 and 2. (TIF) [file pcbi.1011167.s005.tif]

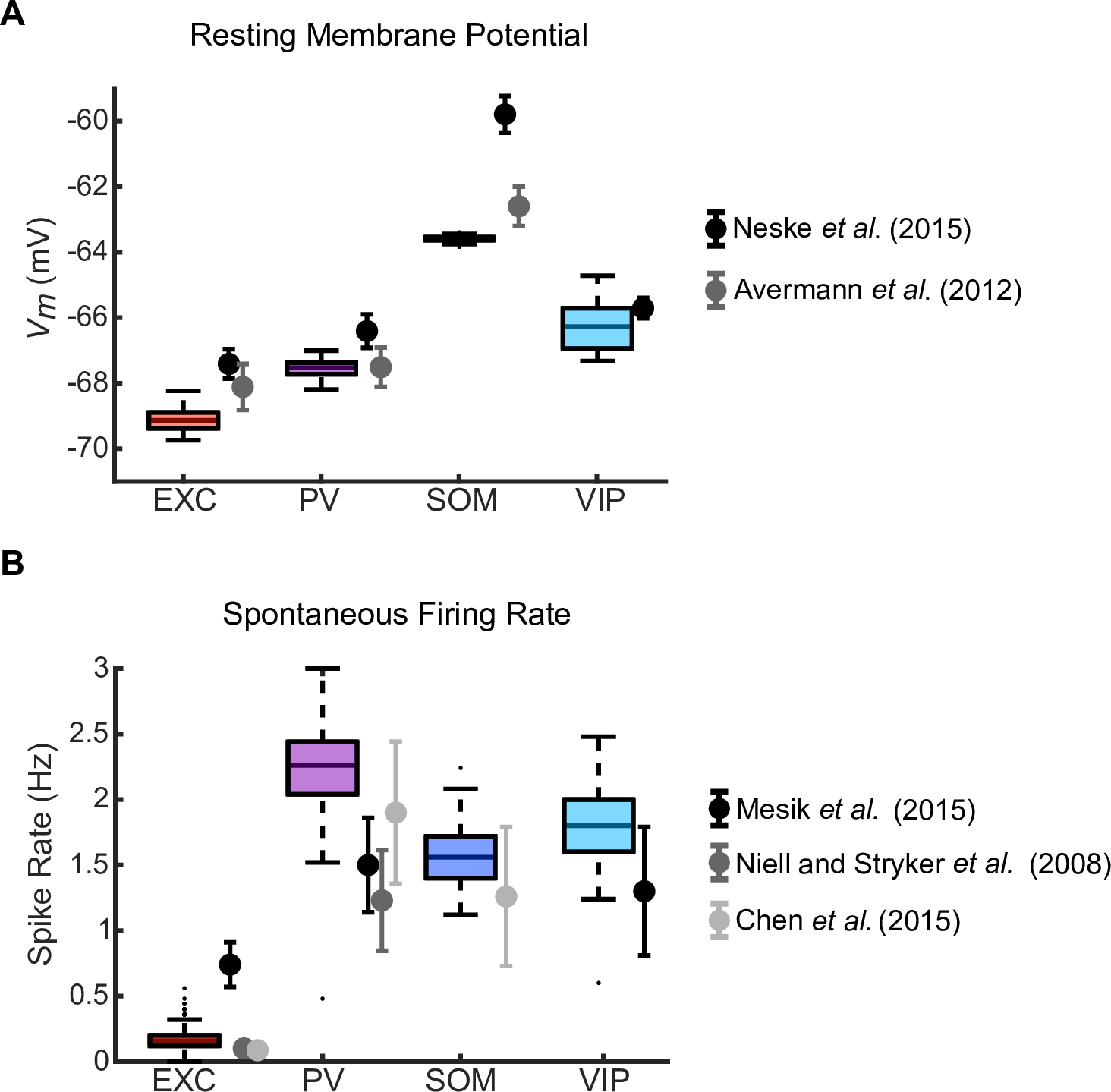

Supplement: S6 Fig — (A) Average resting membrane potential for each neuron subtype in our model over 25 seconds. Our results closely match those found in vivo in L2/3 of the barrel cortex in mice in Neske et al. [65] and Avermann et al. [66]. Results from in vivo data are the mean ± s.e.m. found in those studies. (B) Average spontaneous firing rates in our model over 25 seconds resemble those found in vivo in anesthetized mice in Mesik et al. [63], Neill and Stryker [64], and Chen et al. [62]. Results from in vivo data are the mean ± s.e.m. found in those studies. (TIF) [file pcbi.1011167.s006.tif]

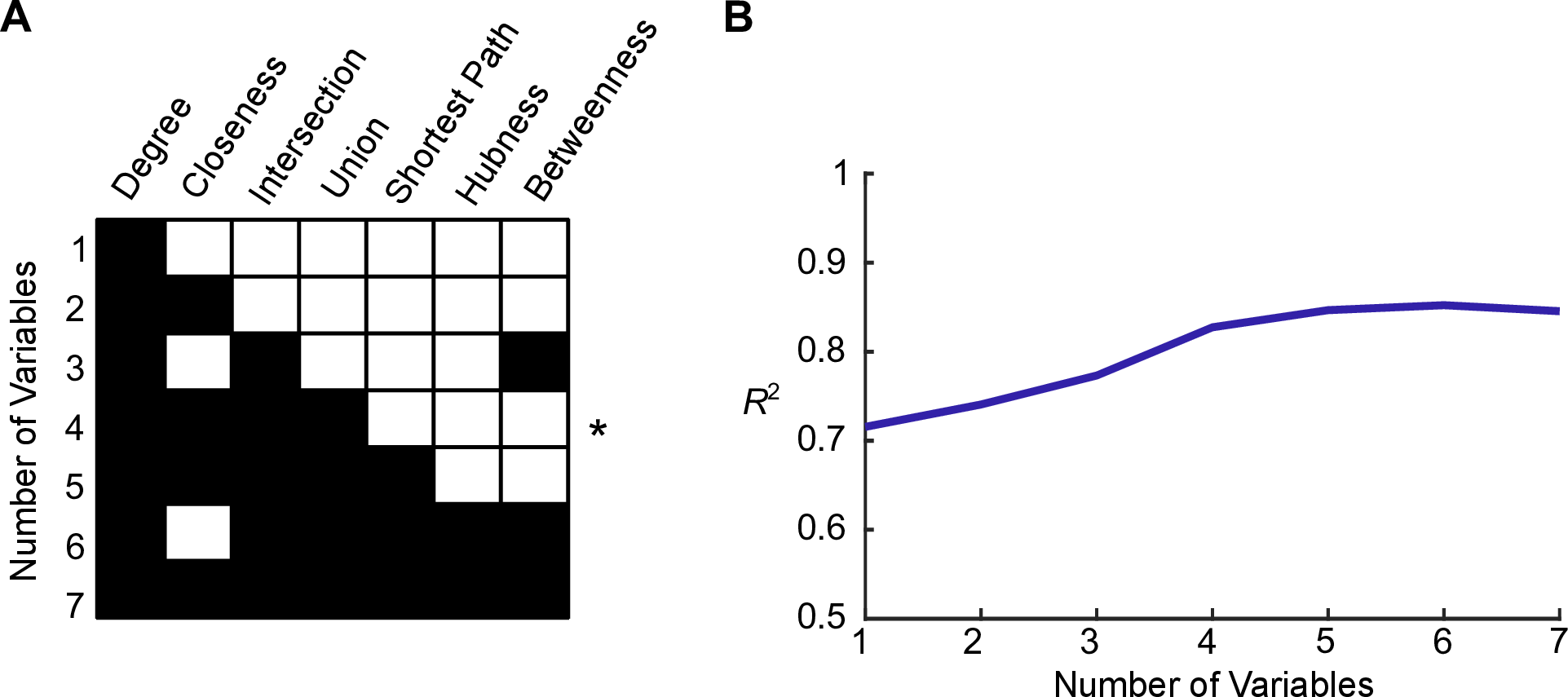

Supplement: S7 Fig — (A) Best subset selection on network variables to predict PCC in ensemble 1. Black boxes indicate which variables were in the best model. * Indicates the final model chosen. (B) The R2 values for each model in (A) plateaus at 4 variables. (TIF) [file pcbi.1011167.s007.tif]

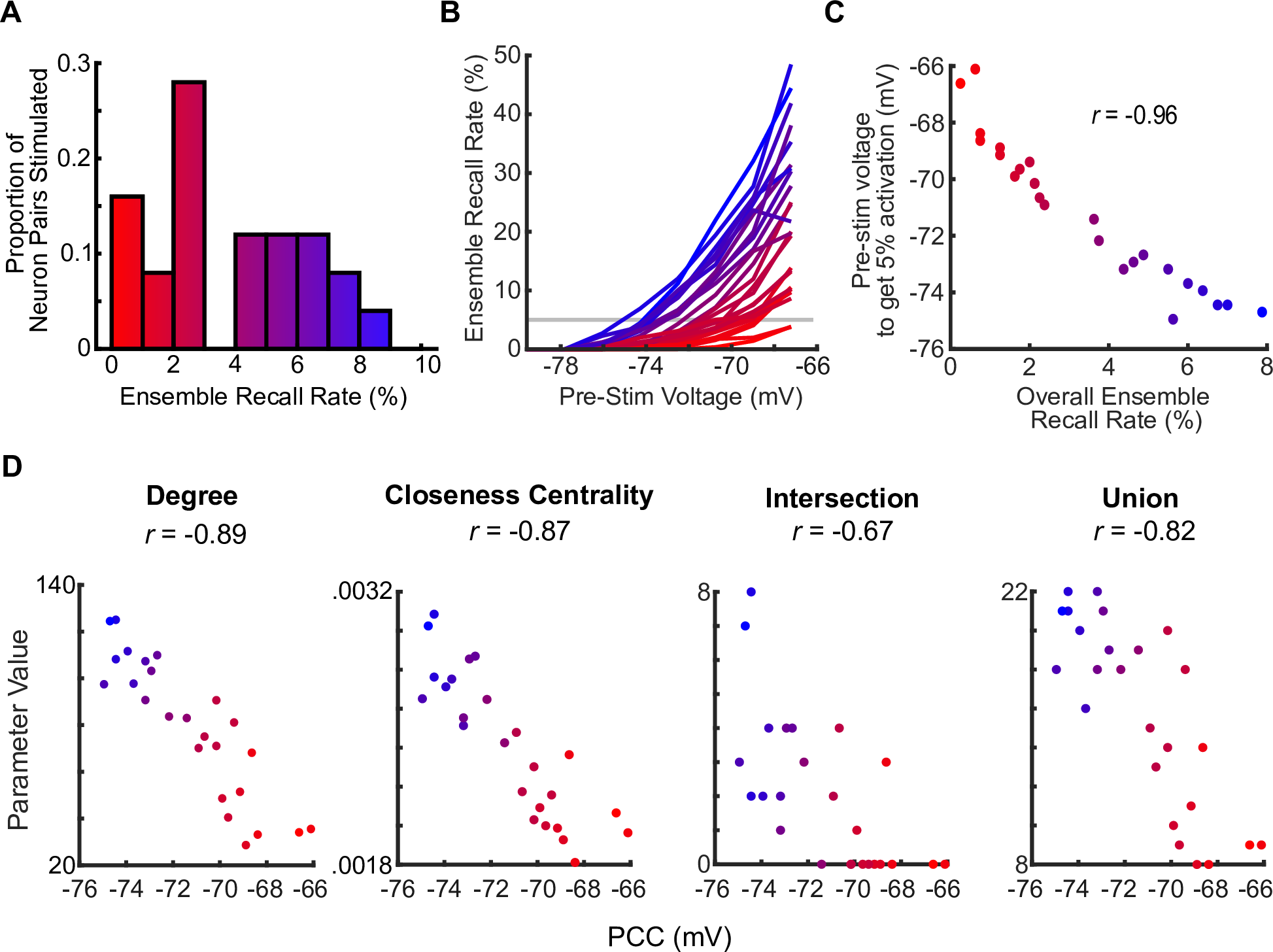

Supplement: S8 Fig — (A) Histogram of ensemble recall rate of 25 different pairs of neurons from ensemble 2. Each pair was stimulated 800 times. We calculated the ensemble recall rate (the fraction of trials where greater than 75% of the ensemble was activated) for each of the 25 neuron pairs. Neuron pairs with higher ensemble recall rates were considered better pattern completion neurons. (B) Probability of ensemble activation increased with the average membrane voltage of ensemble neurons before stimulation of the neuron pair. (C) The pre-stimulation voltage needed for stimulation of a neuron pair to activate an ensemble 5% of the time was significantly and negatively correlated with the overall ensemble recall rate (p = 1.2 × 10^−13^, F-test, n = 25 neuron pairs). Neurons with higher overall ensemble recall rates could achieve a 5% recall rate at voltages farther from threshold. (D) The correlation between each network parameter and the pattern completion capability of each neuron pair. All variables were significantly and negatively correlated with PCC, with degree and closeness centrality having the strongest correlation (from left to right: p = 1.8 × 10^−9^, p = 1.2 × 10^−8^, 2.2 × 10^−4^, 6.6 × 10^−7^, F-test, n = 25 neuron pairs). (TIF) [file pcbi.1011167.s008.tif]

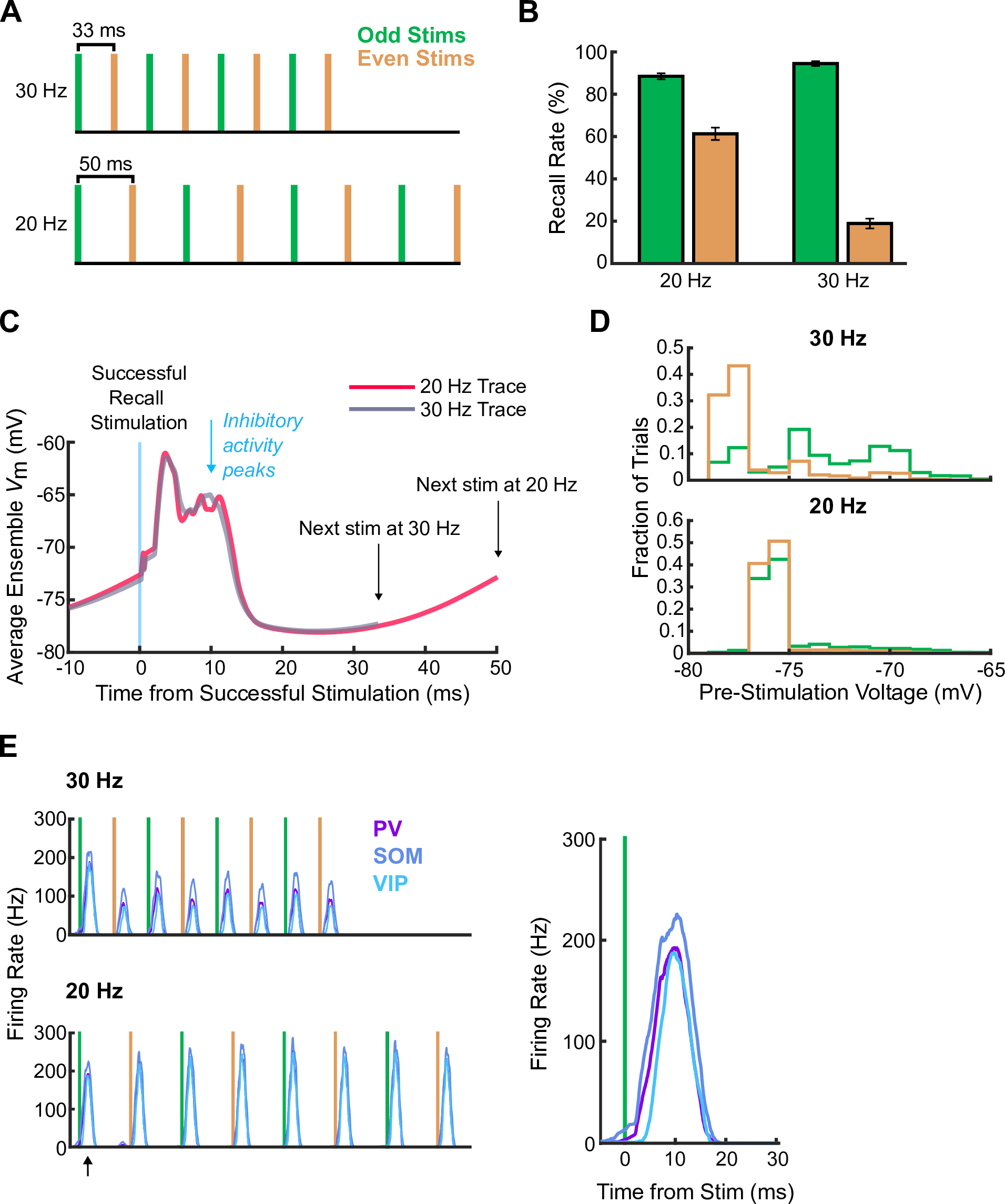

Supplement: S9 Fig — (A) We categorized the 8 stimulations for each stimulation period as either odd or even stimulations. Odd stimulations included the first stimulation of each period and had a higher recall rate compared to even stimulations, as shown in (B). (B) For both 20 Hz and 30 Hz stimulation, odd stimulation events had a higher recall rate than even events. However, the discrepancy between odd and even recall rate was greater at 30 Hz than at 20 Hz. Error bars represent standard error (n = 10 neuron pairs and 100 stimulations for each condition). (C) The average membrane potential of all 36 ensemble neurons during and after a successful ensemble recall event. The blue vertical line indicates the start time of a stimulation that resulted in at least 75% of ensemble neurons firing. In line with inhibitory activity shown in (E), the average ensemble membrane potential decreased abruptly at 10 ms after stimulation. This decrease was followed by a gradual rise in membrane potential back to baseline. However, at 30 Hz, the next stimulation event occurred at 33 ms, so the average ensemble voltage following a successful recall event did not have time to return to baseline. (n = 36 ensemble neurons during 371 recall events at 20 Hz and 132 events at 30 Hz). (D) In agreement with the average membrane potentials shown in (C), histograms of ensemble neuron pre-stimulation voltages at 20 Hz and 30 Hz show that 30 Hz even stimulation events had lower pre-stimulation voltages. This agrees with this group having the lower recall rate. Unlike at 30 Hz, even and odd stimulations at 20 Hz had similar pre-stimulation voltages, and the average was greater than 30 Hz even stimulations. Most pre-stimulation voltages were below the resting membrane potential for excitatory neurons, likely due to delayed inhibitory activity following ensemble events, as shown in (E) (n = 10 neuron pairs and 100 stimulations for each condition). (E) Left: We observed stereotypical delayed inhibitory activity following [file pcbi.1011167.s009.tif]
