# Supplementary material for: Identifying properties of pattern completion neurons in a computational model of the visual cortex
Source: PLoS Comput Biol. 2023 Jun 6;19(6):e1011167. doi: 10.1371/journal.pcbi.1011167 (PMC10275485; doi:10.1371/journal.pcbi.1011167)
Supplement: S1 Table — (PDF) [file pcbi.1011167.s010.pdf]

Supplementary Table 1: Model Information

Ensemble	Number of Neuron Pairs	Regression Coefficients				MSE (mV^2)
		Degree	Closeness Centrality	Intersection	Union	
1	29	-4.60	-1.17	1.88	2.76	0.92
2	25	-3.09	-1.34	0.73	1.60	1.41
