# Supplementary material for: Identifying properties of pattern completion neurons in a computational model of the visual cortex
Source: PLoS Comput Biol. 2023 Jun 6;19(6):e1011167. doi: 10.1371/journal.pcbi.1011167 (PMC10275485; doi:10.1371/journal.pcbi.1011167)
Supplement: S2 Table — (PDF) [file pcbi.1011167.s011.pdf]

Supplementary Table 2: *p*-values for latency to PCC correlation with 100 trials

Sensor 1	Sensor 2	Ensemble 1 *p*-value	Ensemble 2 *p*-value
Model	GCaMP8f	0.0018	5.8×10^{-4}
Model	GCaMP7f	2.7×10^{-16}	3.0×10^{-16}
GCaMP8f	GCaMP7f	1.6×10^{-13}	1.5×10^{-13}
